# Maize *big embryo 6* reveals roles of plastidial and cytosolic prephenate aminotransferases in seed and plant development

**DOI:** 10.1093/plcell/koaf067

**Published:** 2025-06-06

**Authors:** Hui Liu, Jorge El-Azaz, Abou Yobi, Ryo Yokoyama, Shan Wu, Alec D Chin-Quee, Zachary Gorman, Ruthie Angelovici, Anna K Block, Hiroshi A Maeda, Donald R McCarty, Masaharu Suzuki

**Affiliations:** Plant Molecular and Cellular Biology, College of Agricultural and Life Sciences, University of Florida, Gainesville, FL 32611, USA; Department of Botany, University of Wisconsin-Madison, Madison, WI 53706, USA; Department of Biological Sciences, Interdisciplinary Plant Group, University of Missouri, Columbia, MO 65201, USA; Department of Botany, University of Wisconsin-Madison, Madison, WI 53706, USA; Horticultural Sciences Department, College of Agricultural and Life Sciences, University of Florida, Gainesville, FL 32611, USA; Plant Molecular and Cellular Biology, College of Agricultural and Life Sciences, University of Florida, Gainesville, FL 32611, USA; Chemistry Research Unit, US Department of Agriculture-Agricultural Research Service, Gainesville, FL 32611, USA; Department of Biological Sciences, Interdisciplinary Plant Group, University of Missouri, Columbia, MO 65201, USA; Chemistry Research Unit, US Department of Agriculture-Agricultural Research Service, Gainesville, FL 32611, USA; Department of Botany, University of Wisconsin-Madison, Madison, WI 53706, USA; Plant Molecular and Cellular Biology, College of Agricultural and Life Sciences, University of Florida, Gainesville, FL 32611, USA; Horticultural Sciences Department, College of Agricultural and Life Sciences, University of Florida, Gainesville, FL 32611, USA; Plant Molecular and Cellular Biology, College of Agricultural and Life Sciences, University of Florida, Gainesville, FL 32611, USA; Horticultural Sciences Department, College of Agricultural and Life Sciences, University of Florida, Gainesville, FL 32611, USA

## Abstract

In plants, embryo size is determined via interactions between metabolic and developmental signals. Maize (*Zea mays*) *big embryo 6* (*bige6*) enhances embryo size while sharply reducing plant growth. Here, we show that *BigE6* encodes a plastidial prephenate aminotransferase (PPA-AT), a key enzyme in the arogenate pathway for L-phenylalanine (Phe) and L-tyrosine (Tyr) biosynthesis. The maize *BigE6* paralog, *BigE6Like*, encodes a cytosol-localized PPA-AT, revealing Phe and Tyr biosynthesis via cytosolic arogenate as a potential alternative to the known cytosolic phenylpyruvate pathway. Moreover, the single *PPA-AT* gene of Arabidopsis (*Arabidopsis thaliana*) encodes plastidial and cytosolic enzymes by alternative splicing. Transgenic rescue of a *ppa-at* mutant in Arabidopsis demonstrates that the plastidial PPA-AT is indispensable for seed formation due, in part, to its essential role in the female gametophyte. Leaves of *bige6* maize maintained overall homeostasis for aromatic amino acids and downstream metabolites, revealing a resilience of mechanisms that scale growth to a limiting supply of Phe and Tyr. In *bige6* seeds, broad perturbation of amino acid homeostasis is associated with transcriptomic upregulation of growth processes in the embryo and endosperm, implicating amino acid signaling in the regulation of embryo size. Our findings reveal the complexity and developmental dependence of growth responses to limiting amino acid biosynthesis.

## Teaser

Plastidial and cytosolic pathways of aromatic amino acid biosynthesis have partially redundant roles in amino acid homeostasis and growth of seed and plant organs.

## Introduction

In plants, the relative size of embryo and endosperm is a highly heritable trait that affects oil, protein, and starch composition and many other aspects of seed biology (Doehlert and Lambert 1991; Forbis et al. 2002; Miray et al. 2021). While seed size overall is primarily determined by the genotype of the maternal parent (Zhang et al. 2016a), genes expressed zygotically in the filial organs play a substantial, albeit poorly understood, role in controlling partitioning of growth between embryo and endosperm within the seed (Olsen 2020; Li and Yu 2022). Emerging mutants of maize (*Zea mays*) and rice (*Oryza sativa*) are beginning to reveal metabolic and signaling pathways that underlie this critical process (Nagasawa et al. 2013; Suzuki et al. 2015; Mimura et al. 2018; Lee et al. 2019). The *big embryo 6* (*bige6*) mutant of maize described here reveals an unexpected connection between disruption of aromatic amino biosynthesis in the seed and increased embryo growth.

The aromatic amino acids, L-phenylalanine (Phe) and L-tyrosine (Tyr), have essential roles in diverse plant developmental and physiological processes serving as precursors of myriad specialized metabolites and biomass components, as well as protein constituents. Phe, for example, is the precursor of cell wall lignin (Bonawitz and Chapple 2010) as well as diverse phenylpropanoid, flavonoid, and anthocyanin compounds that function in plant defense responses to stress (Ju et al. 1995; Kumar et al. 2020). Tyr-derived metabolites function as electron carriers, antioxidants (Hunter and Cahoon 2007; Nowicka et al. 2021), and defense compounds (Nielsen et al. 2008).

**Figure 1.**
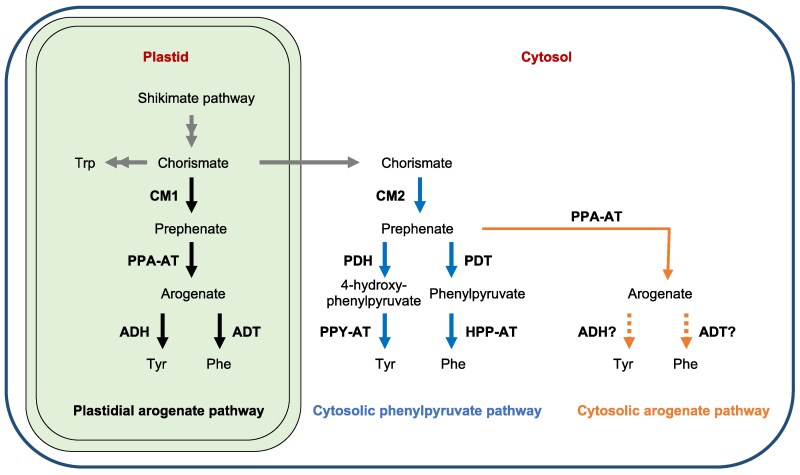
Genetic studies reveal alternative pathways for phenylalanine and tyrosine biosynthesis in plants. CM, chorismate mutase; PPA-AT, prephenate aminotransferase; ADT, arogenate dehydratase; ADH, arogenate dehydrogenase; PDT, prephenate dehydratase; PDH, prephenate dehydrogenase; PPY-AT, phenylpyruvate aminotransferase; HPP-AT, 4-hydroxyphenylpyruvate aminotransferase. Black arrows, reactions of the plastidial arogenate pathway; blue arrows, reactions of the cytosolic phenylpyruvate pathway; orange arrows, reactions of a proposed cytosolic arogenate pathway.

Due to these important roles, Phe and Tyr biosynthetic pathways have been extensively studied. Two alternative pathways for synthesis of Phe and Tyr occur in different cellular compartments of plants that utilize arogenate and phenylpyruvate, respectively, as intermediates (Lynch and Dudareva 2020) (Fig. 1). The plastidial arogenate pathway begins with the conversion of the shikimate pathway product, chorismate, to prephenate by chorismate mutase 1 (CM1). Prephenate is then transaminated to arogenate by prephenate aminotransferase (PPA-AT). Arogenate is in turn converted to Phe and Tyr by arogenate dehydratase (ADT) and arogenate dehydrogenase (ADH), respectively (Cho et al. 2007; Yamada et al. 2008; Maeda et al. 2010; Maeda et al. 2011). In the cytosolic phenylpyruvate pathway, prephenate is synthesized from chorismate by cytosolic chorismate mutase 2 (CM2). Prephenate dehydratase (PDT) catalyzes conversion of prephenate to phenylpyruvate, which is then transaminated to Phe (Fig. 1). Alternatively, Tyr is synthesized by transamination of 4-hydroxyphenylpyruvate formed by prephenate dehydrogenase (PDH) (Schenck et al. 2017; Qian et al. 2019). While the plastidial arogenate pathway is present in all plants, the phylogenetic distribution of the cytosolic phenylpyruvate pathway has not been established. Phe synthesis from phenylpyruvate has been characterized in petunia (*Petunia* × *hybrida*) petals that produce large amounts of Phe-derived volatiles (Yoo et al. 2013; Qian et al. 2019). Synthesis of Tyr via phenylpyruvate is apparently restricted to legumes that have a cytosolic, feedback-insensitive PDH enzyme (Rubin and Jensen 1979; Schenck et al. 2017). Therefore, the plastidial arogenate pathway is believed to be the predominant source for Phe and Tyr biosynthesis (Maeda et al. 2010; Maeda et al. 2011; Schenck and Maeda 2018; Lynch and Dudareva 2020).

While the arogenate pathway is known to be active in plastids, detailed analyses of substrate specificities and subcellular localizations of pathway enzymes in model plants reveal a more complex picture of Phe and Tyr biosynthesis. Plants typically have 2 isoforms of CM, the enzyme that converts chorismate to prephenate, CM1 localized in plastid and CM2 in cytosol (Goers and Jensen 1984; Singh et al. 1986; Poulsen and Verpoorte 1991; Eberhard et al. 1996; Lynch 2022). The widespread occurrence of cytosolic CM2 in plants suggests a broader role for prephenate metabolism in cytosol. Studies in Arabidopsis confirm CM2 involvement in Phe biosynthesis in vivo (Qian et al. 2019). Arabidopsis has 6 ADT enzymes. Of these, ADT1, ADT2, and ADT6 have both ADT and PDT activities, whereas ADT3, ADT4, and ADT5 have only ADT activity (Cho et al. 2007). Subcellular localization studies show that monofunctional ADT3 that lacks PDT activity is detected in both cytosol (Para et al. 2016) and plastids (Bross et al. 2017). These findings imply that some enzymes of the arogenate pathway, if not a complete pathway, are present in the cytosol.

Biochemical and genetic evidence highlights the essential roles of Phe and Tyr derived from the arogenate pathway in plant growth and development (Corea et al. 2012; Chen et al. 2016; El-Azaz et al. 2018; de Oliveira et al. 2019). For example, disruption of maize *ADT2* causes defects in chloroplast morphology, a dwarf phenotype, and increased susceptibility to *Ustilago maydis* infection (Ren et al. 2024). Moreover, the Tyr-deficient *adh1* mutant of maize (*arodh1*) inhibits synthesis of zein storage proteins in the seed endosperm and causes reallocation of amino acids between embryo and endosperm (Holding et al. 2010). By contrast, disruption of the phenylpyruvate pathway has little discernible impact on plant morphology (Qian et al. 2019; Schenck et al. 2020). Despite extensive research on functions of Phe and Tyr pathways, we thus far have limited insight into relative contributions of arogenate and phenylpyruvate pathways in plant and seed development.

Here, we show that ablation of plastid-localized PPA-AT in the *bige6* mutant of maize has pleiotropic effects on embryo size, zein storage protein accumulation, plant growth rate, and amino acid metabolism. Key seed phenotypes including increased embryo size and elevated synthesis of Phe-derived anthocyanins in aleurone are strikingly counterintuitive. We attribute viability of *bige6* plants to presence of a paralog, *BigE6Like*, that encodes a cytosol-localized PPA-AT, which enables cytosolic synthesis of arogenate. We further demonstrate that in Arabidopsis plastidial and cytosolic forms of PPA-AT are generated by alternative splicing of a single *PPA-AT* gene, suggesting that the capacity to synthesize arogenate in the cytosol is broadly conserved in flowering plants. Transgenic rescue experiments using a *ppa-at* knockout created by *CRISPR/Cas9* editing demonstrate that plastidial PPA-AT specifically is indispensable for female gametophyte function in Arabidopsis. The paradoxical large embryo phenotype of maize *bige6* is associated with an extensive perturbation of amino acid homeostasis in the seed combined with massive upregulation of growth-related processes including genes for ribosome assembly, RNA processing, and protein synthesis coupled with downregulation of autophagy genes. Together, our results provide insights into complex roles of plastid and cytosol pathways for Phe and Tyr synthesis in seed and plant development.

## Results

### The *bige6* mutant has a larger embryo but a dramatically reduced plant growth rate

**Figure 2.**
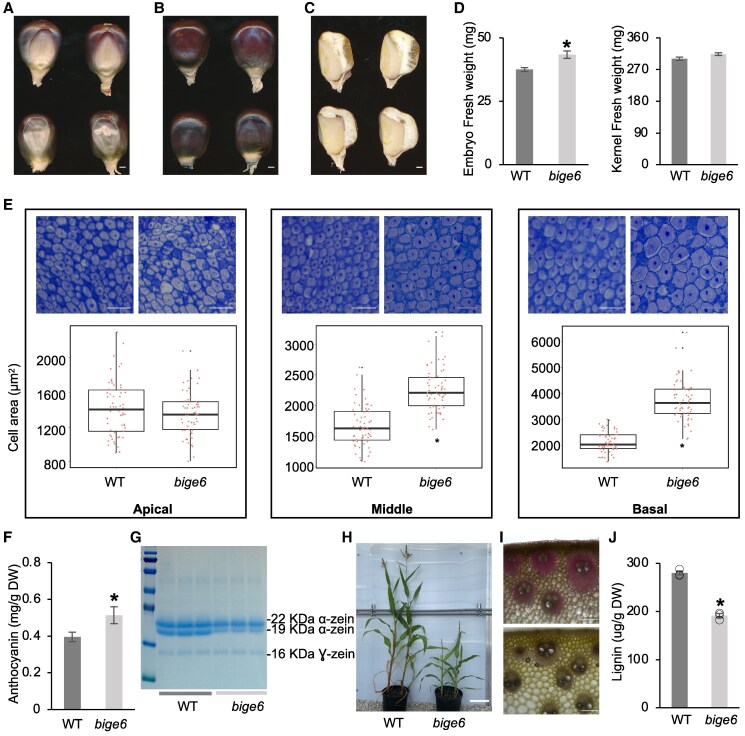
The *bige6* mutant has pleiotropic kernel and plant phenotypes. **A)** Germinal views of WT (upper row) and *bige6* (lower row) kernels detached from heterozygous segregating ears. Scale bar = 1 mm. **B)** Abgerminal views of WT (upper row) and *bige6* (lower row) mature dry kernels. Scale bar = 1 mm. **C)** Longitudinal hand section of WT (upper row) and *bige6* mutant (lower row) mature kernels. Scale bar = 1 mm. **D)** Fresh weights of excised embryos (left) and whole kernels (right) of WT (dark gray) and *bige6* mutant (light gray) harvested at 28 DAP. Ten independent samples were measured for each genotype. **E)** Histological analysis of embryo scutellum of WT and *bige6* mature kernels at 28 DAP. Upper panels show representative images highlighting apical, middle, and basal regions of the scutellum in embedded thin sections of WT (left) and *bige6* (right) embryos. Box plots of cell size in scutellum regions of WT and *bige6* embryos. At least 20 cells were counted per region. Scale bar = 100 *μ*m. Center horizontal line indicates medians; box limits are shown with upper and lower quartiles; whiskers show 1.5 × interquartile range; points outside the plot indicated outliers. **F)** Anthocyanin contents of WT and *bige6* mature kernels. Nine independent kernels for each genotype were used for the quantitation. DW, dry weight. **G)** SDS–PAGE analysis of zein proteins extracted from mature WT and *bige6* kernels. Three major bands correspond to 16-, 19-, and 22-kDa zeins. **H)** WT and *bige6* plants at time of flowering. Plants were grown from seeds that were sown on the same day. Scale bar = 20 cm. **I)** Phloroglucinol–HCl staining of cross sections from the 8th internode of WT (upper panel) and *bige6* (lower panel) plants. Scale bar = 100 *μ*m. **J)** Total lignin content of WT and *bige6* 8th internodes assayed using the acetyl bromide method (Moreira-Vilar et al. 2014). Three independent plants were used for each genotype. Values are means ± SE. *Differences with *t* test *P* < 0.05.

To determine the genetic and molecular basis of variation in relative embryo size in maize, we screened the UniformMu transposon population (McCarty et al. 2005) for embryo size phenotypes (Suzuki et al. 2015; Mimura et al. 2018). Genetic complementation tests confirmed 3 independent *bige6* alleles, *bige6-umu1, bige6-umu2,* and *bige6-umu3*, that have indistinguishable phenotypes (Fig. 2 and Supplementary Fig. S1). In *bige6* kernels, like previously described *bige1* (Suzuki et al. 2015), the area of embryo visible on the germinal face of the W22 kernels was enlarged relative to wild type (WT), while the total sizes of mutant and WT kernels were comparable (Fig. 2A and Supplementary Fig. S1A and B). In addition to having altered relative size of embryo, in the color-converted W22 inbred background the aleurone of *bige6* endosperm was more intensely pigmented compared with WT (Fig. 2B). Sagittal sections of mature kernels indicated *bige6* embryos have an enlarged scutellum compared with WT (Fig. 2C). At maturity, the fresh weight of mature *bige6* embryos was 16% greater than that of WT embryos, whereas whole kernel fresh weights of WT and mutant were similar (Fig. 2D). In developing kernels, an increase in embryo fresh weight was detected by 28 days after pollination (DAP) (Supplementary Fig. S1C). To determine the cellular basis for the enlarged scutellum of *bige6* mutant embryos, we performed histological analysis using thin sections of WT and mutant embryos. We compared distributions of cell size in apical, middle, and basal regions of the embryo scutellum at maturity (Supplementary Fig. S1D). Average cell size of *bige6* embryo scutellum was increased in the middle and basal regions of the tissue, indicating that enlargement of the *bige6* embryo is at least partly due to increased cell size in those regions (Fig. 2E). Consistent with increased cell size, ploidy levels of nuclei extracted from *bige6* embryos were also slightly elevated indicating a modest increase in endopolyploidy (Supplementary Fig. S1E). By contrast, cell size in the apical region of scutellum was not affected in the *bige6* mutant embryo (Fig. 2E), indicating that *bige6* effects on cell size were not uniform across regions of the scutellum. Quantification of seed anthocyanins confirmed that mutant kernels had 30% greater anthocyanin content (Fig. 2F). Moreover, *bige6* kernels lacked vitreous endosperm (Fig. 2C and Supplementary Fig. S1A and B) indicative of an opaque phenotype typically associated with altered protein and/or starch accumulation (Kim et al. 2006; Holding et al. 2010; Miclaus et al. 2011; Myers et al. 2011; Wang et al. 2014; Zhang et al. 2018). Similar to classical opaque endosperm mutants (Zhang et al. 2018), *bige6* kernels contained reduced amounts of 19- and 22-kDa zein proteins as well as 16-kDa γ-zein (Fig. 2G). By contrast, SDS–PAGE analysis of nonzein proteins in WT and *bige6* kernels did not detect marked differences in accumulation of specific proteins (Supplementary Fig. S1F). In addition, we measured starch content of WT and *bige6* kernels. Starch contents of WT and *bige6* kernels were comparable on a dry weight basis (*bige6* 53.95% ± SD 1.14 vs. WT 52.11% ± SD 1.89, *P* = 0.17). To determine whether oil content was altered, single-kernel near-infrared (skNIR) spectroscopy analysis (Jiang et al. 2007; Gustin et al. 2013) was performed to compare estimated oil composition in WT and *bige6* kernels. While *bige6* mutant embryos are enlarged, oil content was reduced in the mutant by 21.9% (*bige6* 4.73% ± SD 0.43 vs. WT 6.06% ± SD 0.47, *P* = 4.87065E^−10^).

Homozygous *bige6* kernels germinated slowly with reduced frequency, and though viable mutant plants small in stature compared with WT with short, narrow, slightly pale green leaves. Flowering time of *bige6* plants did not differ from WT (Fig. 2H and Supplementary Fig. S1G and H). These phenotypes indicated that in contrast to *bige1* (Suzuki et al. 2015) timing of differentiation of leaves and reproductive organs was not altered in *bige6*. Although small, *bige6* plants were both male and female fertile. Overall, the *bige6* altered growth but did not affect developmental patterning of the plant body or fertility. In addition to having reduced stature, we noticed that mutant plants were physically more pliable than WT indicating reduced stiffness. Consistent with this phenotype, phloroglucinol–HCl staining of the internode cross sections from WT and *bige6* mutant plants revealed a marked decrease in lignin content of *bige6* mutant stems (Fig. 2I). Quantification of total lignin using the acetyl bromide method (Moreira-Vilar et al. 2014) confirmed a roughly 33% reduction of total acetyl bromide soluble lignin in the mutant per dry weight basis in the mutant (Fig. 2J).

### The *BigE6* gene encodes a PPA-AT

**Figure 3.**
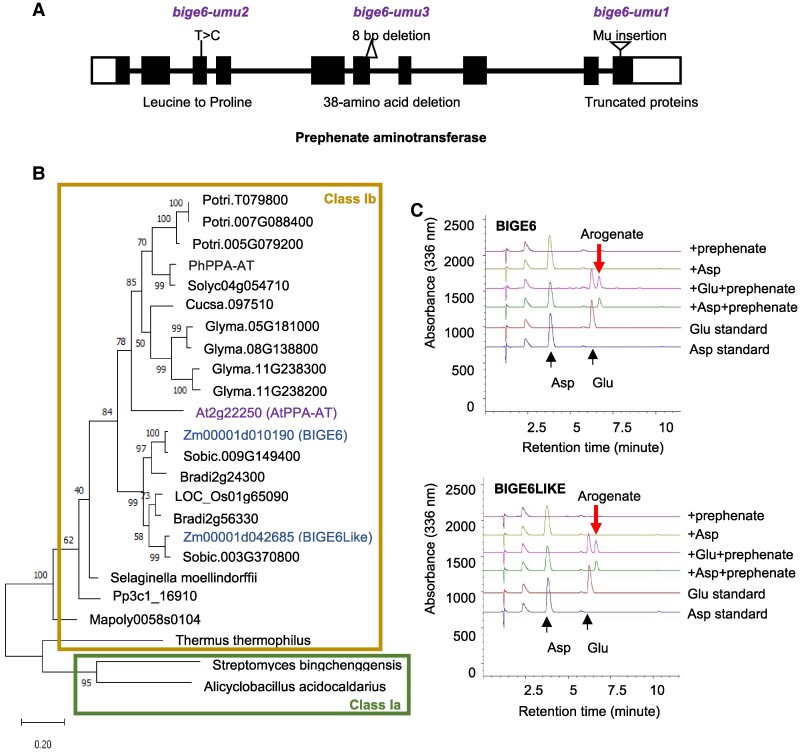
*BigE6* and *BigE6Like* genes encode putative PPA-ATs. **A)** Gene structure of *BigE6* and 3 mutant alleles. Black boxes and white boxes represent coding exons and UTRs, respectively. The black line represents introns. **B)** Phylogenetic tree of BIGE6 and BIGE6LIKE together with representative Ia and Ib class aspartate aminotransferases from diverse species. The tree was constructed using the maximum likelihood method in MEGA version 11 with a bootstrap value of 1,000. **C)** In vitro PPA-AT enzyme assays of recombinant His-tagged BIGE6 and BIGE6LIKE. Enzyme activities of BIGE6 (upper row) and BIGE6LIKE (lower row) were analyzed using L-aspartate (Asp), or L-glutamic acid (Glu) as amino donors and prephenate as acceptor substrate.

Mu-seq analysis (McCarty et al. 2013) of the *bige6*-*umu1* allele identified a candidate causative Mutator (Mu) transposon insertion in the 10th exon of *Zm00001d010190* (Fig. 3A), a gene encoding a putative PPA-AT involved in the arogenate pathway of Phe and Tyr biosynthesis (Graindorge et al. 2010; Maeda et al. 2010; Maeda et al. 2011; Graindorge et al. 2014). RT-PCR analysis of leaves using primers spanning the Mu insertion site detected no transcripts of *bige6-umu1* (Supplementary Fig. S2A). The Mu insertion in *Zm00001d010190* is predicted to cause truncation of 34 amino acids from the C-terminal end of the PPA-AT protein. Genomic sequences of 2 additional alleles, *bige6-umu2* and *bige6-umu3*, revealed a single-nucleotide substitution in *bige6-umu2* and an 8-bp deletion in *bige6-umu3*, respectively, confirming the identification of the *BigE6* gene (Fig. 3A). The altered transcripts encoded by *bige6-umu2* and *bige6-umu3* alleles were expressed at levels compared with WT (Supplementary Fig. S2A).

The single-nucleotide substitution in *bige6-umu2* converts a highly conserved Leu at position 127 to Pro (Maeda et al. 2011). The L127P substitution located in an alpha helix (Holland et al. 2018) is likely to disrupt protein structure by preventing the intrahelix hydrogen bonding (Kim and Kang 1999). To further assess the structural impact of Leu-127, we used the Arabidopsis PPA-AT (AtPPA-AT) crystal structure (Holland et al. 2018) to model the impact of the corresponding L141P mutation using the DynaMut web server (Rodrigues et al. 2018). The results confirmed the prediction that the L127P substitution is likely to destabilize the alpha helix (Supplementary Fig. S2B). Consistent with the predicted loss of protein stability, expression of the recombinant *bige6-umu2* protein fused with His tag in *Escherichia coli* produced less protein compared with WT (Supplementary Fig. S2C). The deletion in *bige6*-*umu3* spanning the junction between 6th intron and 6th exon is predicted to disrupt mRNA splicing. We sequenced RT-PCR products derived from total RNA isolated from *bige6-umu3* homozygous embryos and confirmed that the spliced transcript contained an in-frame deletion of 38 amino acids in the predicted mutant BIGE6 protein (Supplementary Fig. S2D). The deleted region is highly conserved among plant PPA-AT proteins and includes several amino acids that are likely essential for enzyme activity (Maeda et al. 2011; Holland et al. 2018). As noted above, 3 *bige6* alleles have indistinguishable seed and plant growth phenotypes. The indistinguishable phenotypes together with molecular analyses of the 3 alleles are consistent with all being null mutants for PPA-AT.

### BIGE6 and BIGE6LIKE paralogs both exhibit PPA-AT activity

The viability of *bige6* mutant plants suggested a possibility of genetic redundancy for *PPA-AT* in maize. This contrasts with the presence of a single *PPA-AT* gene (Graindorge et al. 2010; Maeda et al. 2011) in Arabidopsis, which is predicted to be essential for completing the plant's life cycle (Pagnussat et al. 2005). Consistent with the predicted genetic redundancy in maize, phylogenetic analyses of *PPA-AT* genes from representative plant genomes (Fig. 3B) revealed a closely related paralog in the maize genome (*Zm00001d042685*), here designated as *BigE6Like*. BIGE6 and BIGE6LIKE likely diverged within monocots (Fig. 3B). Genomes of angiosperms vary in the number of *PPA-AT* genes. Notably, *Arabidopsis thaliana* and *O. sativa* have a single *PPA-AT* gene, whereas maize and *Glycine max* genomes have 2 and 4 genes, respectively (Fig. 3B). BIGE6LIKE shared 88% of amino acid sequence identity with BIGE6 and had all conserved domains found across plant PPA-ATs (Supplementary Fig. S3), suggesting that BIGE6LIKE is a functional PPA-AT.

To experimentally test whether BIGE6 and BIGE6LIKE have PPA-AT activity, we generated and purified their recombinant proteins and conducted in vitro enzyme assay. As shown in Fig. 3C, both BIGE6 and BIGE6LIKE recombinant proteins were capable of producing arogenate from prephenate in the presence of an amino donor, glutamate or aspartate. This was not the case in the reactions lacking prephenate or the amino donor (Fig. 3C). Consistent with genetic redundancy of maize *PPA-ATs*, public gene expression databases (https://www.maizegdb.org/) indicated that both *PPA-AT* genes, *BigE6* and *BigE6Like*, are broadly expressed in maize tissues including both embryo and endosperm. In the seed, *BigE6* is preferentially expressed in the embryo, whereas *BigE6Like* is more highly expressed in endosperm (Chen et al. 2014; Walley et al. 2016, Supplementary Fig. S4). These results support the hypothesis that viability of *bige6* mutant plants is due to the remaining PPA-AT activity derived from BIGE6LIKE.

### BIGE6 and BIGE6LIKE are targeted to the plastids and cytosol, respectively

**Figure 4.**
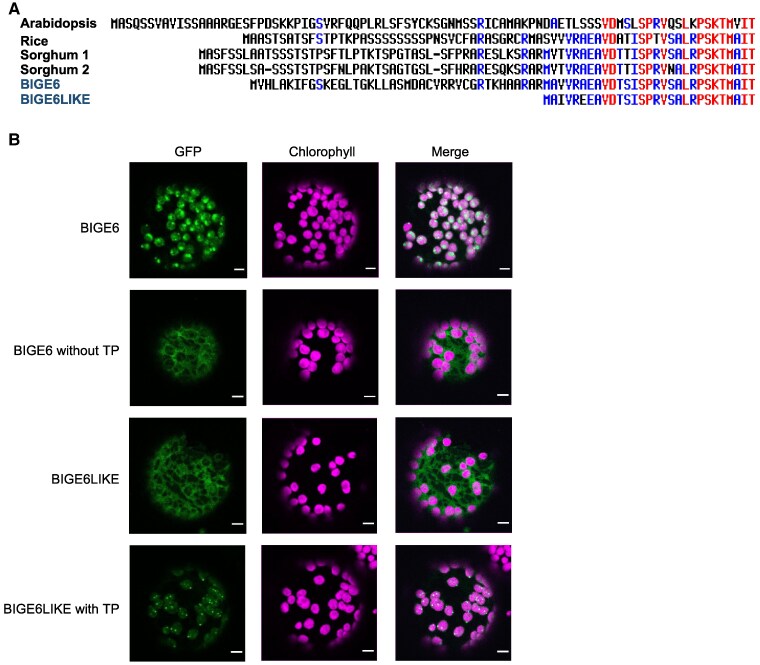
BIGE6 and BIGE6LIKE are localized to plastids and cytosol, respectively. **A)** Sequence alignment of PPA-AT N-terminal regions. **B)** Subcellular localization of BIGE6, truncated BIGE6 with TP removed, BIGE6LIKE, BIGE6LIKE including N-terminal TP fusion. All proteins were expressed as C-terminal GFP fusions in *N. benthamiana* mesophyll protoplasts. Chlorophyll autofluorescence (magenta) was used as a marker for chloroplast. Scale bars indicate 10 *μ*m.

Previous studies have shown that PPA-AT proteins contain an N-terminal plastid transit peptide (TP) (Maeda et al. 2010) and are targeted to the plastids (Dal Cin et al. 2011). However, amino acid sequence alignments of BIGE6 and BIGE6LIKE with PPA-AT proteins from other plant species (Fig. 4A and Supplementary Fig. S3) highlighted an apparent lack of the N-terminal TP in BIGE6LIKE. To rule out the possibility that an alternative transcription start site in the *BigE6Like* gene produces an mRNA incorporating a TP, RT-PCR products were sequenced to confirm the 5′UTR of *BigE6Like* gene, which was consistent with the B73v4 gene model (MaizeGDB). We further analyzed flanking genomic sequence up to 5 kb upstream of the 5′UTR. We found no upstream ATG start codons that could be spliced to incorporate a TP. In addition, analysis of maize RAMPAGE and CAGE data (Mejía-Guerra et al. 2015; Mejia-Guerra et al. 2018) indicated that all detected *BigE6Like* transcription start sites are consistent with transcripts that encode a protein lacking an N-terminal TP. Furthermore, BIGE6 is included in the maize plastid stroma proteome, whereas BIGE6LIKE is not detected in the plastids (http://ppdb.tc.cornell.edu/) (Majeran et al. 2012). Taken together, these results suggested that BIGE6LIKE is likely localized in the cytosol.

To confirm the subcellular localization of 2 maize PPA-ATs, we transformed *Nicotiana benthamiana* leaf cells with *BigE6* and *BigE6Like* coding sequences fused with eGFP (hereafter GFP) gene at the C terminus using Agrobacterium infiltration. Protoplasts of transformed cells were isolated and analyzed for GFP subcellular localization using a fluorescence microscope (Yoo et al. 2007). GFP fluorescence was detected in the plastids of cells transformed with the BIGE6-GFP expression vector. Interestingly, similar to the localization of *Solanum lycopersicum* PPA-AT (Dal Cin et al. 2011), the BIGE6-GFP signal was detected in a punctate pattern that overlaps with chlorophyll autofluorescence. By contrast, GFP signal was detected in the cytosol of cells transformed with the BIGE6LIKE-GFP vector. When the N-terminal region of BIGE6 was attached to BIGE6LIKE-GFP, the fusion protein was localized in the plastids (Fig. 4B). These results indicated that while BIGE6 and BIGE6LIKE have similar enzyme activities (Fig. 3C), they are localized to distinct compartments, plastids and cytosol, respectively.

### A cytosolic PPA-AT is encoded by alternative splicing in Arabidopsis

**Figure 5.**
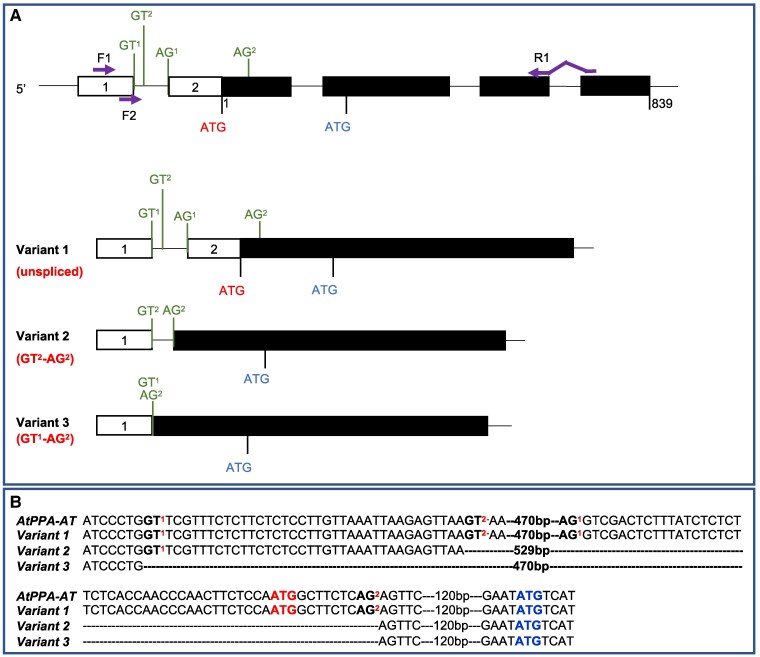
Alternative splicing variants of *AtPPA-AT* encode plastidial and cytosolic isoforms. **A)** Schematic structure of *AtPPA-AT* gene and 3 transcript variants detected by RT-PCR in total RNA from root. GT^1^, GT^2^, AG^1^, and AG^2^ indicate potential alternative splice sites. The translation start codons are shown in red for plastidial PPA-AT and blue for cytosolic isoforms, respectively. Purple arrows are primer pairs used for RT-PCR analysis. Positions of nucleotide positions are referred from the first start codon. Black boxes and white boxes represent coding exons and UTRs, respectively. The thin black lines between boxes represent introns. **B)** Alignments of splice variant DNA sequences with *AtPPA-AT* genomic sequence. Dotted lines indicate gaps of nucleotide sequences.

The above identification of plastidial and cytosolic maize PPA-AT isoforms encoded by separate genes raises a question whether other plants such as Arabidopsis with a single *PPA-AT* gene have only the plastidial isoform. However, we noted that there are at least 3 predicted alternative transcript models of *AtPPA-AT* annotated at NCBI: NM_127791.3, NM_001036317.1, and NM_179691.4. The first 2 encode a predicted plastidial form of AtPPA-AT, whereas the third is an alternatively spliced transcript that lacks the N-terminal TP and is therefore predicted to encode a cytosolic enzyme (Supplementary Fig. S5A). To experimentally confirm the structures of the predicted splicing variants, we performed RT-PCR using total RNA from Arabidopsis roots (Supplementary Fig. S5B) where the *AtPPA-AT* expression is relatively high (https://www.arabidopsis.org). The amplified products by RT-PCR were sequenced. Collectively, we detected 3 distinct splicing variants, 2 of which match NCBI transcript models. In Variant 1, the intron located in the 5′UTR is unspliced resulting in a plastid-targeted protein, whereas the second, Variant 3, encodes a putative cytosolic form of the enzyme. However, we did not detect variants that utilized the AG^1^ splice site predicted in NM_179691.4. Instead, we detected a novel splice junction at GT^2^–AG^2^ that removes the first in frame ATG (Variant 2) that would incorporate the TP (Fig. 5). Hence, 2 of 3 validated splicing variants encode predicted cytosolic proteins. To test subcellular localizations of 3 variants, we fused their coding regions with GFP and expressed them in *N. benthamiana* cells. The GFP signals confirmed that Variant 1 is localized in the chloroplast, while Variant 2 and Variant 3 without TPs are localized in the cytosol (Supplementary Fig. S5C and D). These results revealed that the Arabidopsis *PPA-AT* gene has capacity to undergo alternative splicing to generate transcript variants encoding both plastidial and cytosolic PPA-ATs.

### The plastidial PPA-AT isoform is required for female gametophyte function and seed formation in Arabidopsis

**Figure 6.**
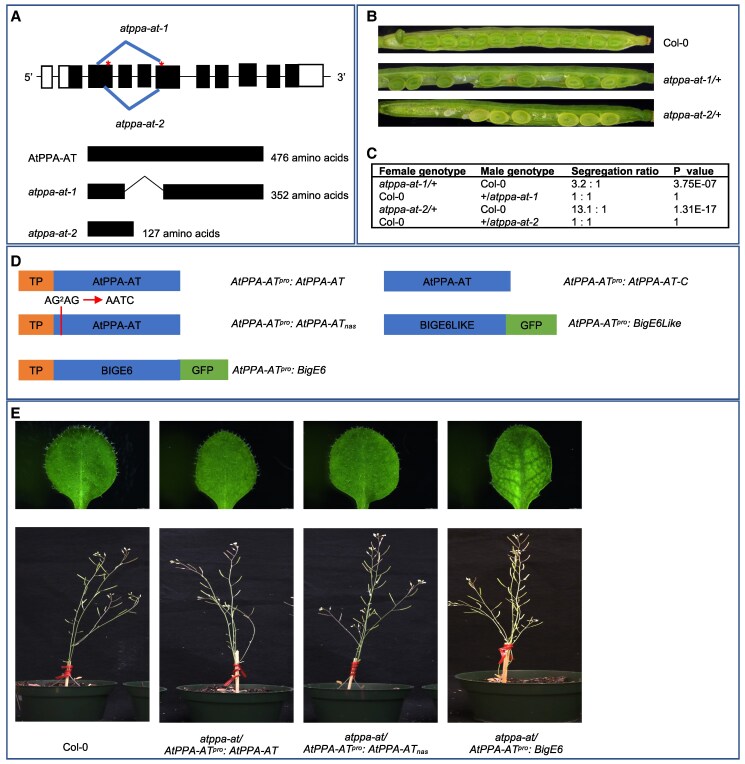
Arabidopsis *ppa-at* mutants generated by *CRISPR/Cas9* are rescued by transgenes expressing the plastidial PPA-AT isoform, but not cytosolic PPA-AT. **A)** Genomic structure of WT *AtPPA-AT* and 2 deletion alleles of the mutants generated by *CRISPR/Cas9*. The asterisks indicate gRNA target sites. The lower panel shows predicted translated protein products derived from WT and 2 mutant alleles. Black boxes and white boxes represent coding exons and UTRs, respectively. The thin gray lines between boxes represent introns. **B)** Seed set in developing siliques of Col-0 and *atppa-at* heterozygotes. **C)** Segregation ratios of reciprocal crosses between Col-0 and *atppa-at*/+ parents. **D)** Schematic of transgenes tested for ability to rescue the *atppa-at-2* mutant. All transgenes were driven by the native Arabidopsis *PPA-AT* promoter (1,825-bp upstream region from the translation start site). **E)** Leaf and plant phenotypes of *atppa-at-2* plants rescued by Arabidopsis *PPA-AT*, *AtPPA-ATnas* (nonalternative splicing [nas]) and maize *BigE6*.

The results shown above suggest that the capacity for expressing plastidial and cytosolic forms of PPA-AT is broadly distributed in angiosperm genomes, raising a question of whether one or both forms are essential for plant development. To create a platform for functional analysis of plastidial and cytosolic forms of PPA-AT, we ablated the single *ppa-at* gene of Arabidopsis (*AtPPA-AT)* using an egg-specific *CRISPR/Cas9* gene editing strategy (Wang et al. 2015). We isolated 2 independent deletion alleles. The *atppa-at-1* allele has an in-frame deletion which is predicted to cause deletion of 124 amino acids. The *atppa-at-2* allele generates a premature stop codon, leading to a large deletion in the C terminus (Fig. 6A). Heterozygous *atppa-at/+* mutant plants formed partially filled siliques containing a large number of empty ovule positions. In addition, shriveled, defective seeds were occasionally observed (Fig. 6B). We genotyped seedlings grown from normal seeds harvested from heterozygous siliques and found that among the progeny heterozygotes were underrepresented, whereas no homozygous *atppa-at-2* mutant seedlings were detected (WT:heterozygote = 1.6:1). Therefore, *atppa-at* mutants are homozygous lethal during early seed development and/or disrupt function of the female gametophyte. To test transmission of *atppa-at* mutations through male and female gametophytes, we made reciprocal crosses between *atppa-at* heterozygous plants and WT. As shown in Fig. 6C, *atppa-at* mutant alleles failed to transmit through the female. This result was consistent with detection of an *atppa-at* mutant in a large-scale genetic screen for gametophyte mutants (Pagnussat et al. 2005). Together, these results indicated that *PPA-AT* is essential for female gametophyte function, while, somewhat surprisingly, the loss of *PPA-AT* does not perturb male gametophyte transmission in Arabidopsis plants.

Next, we asked whether a transgene expressing cytosolic and plastidial PPA-AT could rescue the *atppa-at* null mutant. As a positive control, we constructed a transgene carrying full-length protein coding cDNA for Arabidopsis PPA-AT fused downstream of a 1,825-bp *PPA-AT* promoter that included the 5′ untranslated region (UTR). The resulting *AtPPA-AT^pro^:AtPPA-AT* transgene has potential to encode both plastidial and cytosolic forms (Fig. 6D). We transformed *atppa-at*-*2* heterozygous plants with *AtPPA-AT^pro^:AtPPA-AT* by floral dip transformation (Clough and Bent 1998). Analysis of segregating T_2_ progeny identified 3 phenotypically normal homozygous *atppa-at*-*2* mutant lines that carried the transgene, indicating that the expression of the full length of *AtPPA-AT* coding sequence was able to fully rescue the mutant (Fig. 6E). Next, we tested *AtPPA-AT^pro^:AtPPA-AT_nas_*, a transgene in which a silent mutation in splice acceptor site AG^2^ has the capacity to encode the plastidial form but not the alternatively spliced cytosolic form (Fig. 6D). Similar to the control experiment, 3 independently rescued *atppa-at-2* homozygous mutant lines were successfully isolated, indicating that the plastidial form of AtPPA-AT alone is sufficient to complement loss of *PPA-AT* function in Arabidopsis (Fig. 6E). Additionally, the expression of a cDNA encoding the plastid-targeted maize BIGE6 driven by the same native *PPA-AT* promoter of Arabidopsis successfully rescued the *atppa-at-2* mutant (Fig. 6D). This result not only confirmed that plastidial form of PPA-AT is sufficient to complement the *atppa-at* mutant but also established that maize BIGE6 is a functional ortholog of Arabidopsis PPA-AT. Interestingly, however, while AtPPA-AT rescued plants were indistinguishable from WT, the BIGE6 rescued plants had a reticulated leaf phenotype, similar to the Tyr-deficient mutant phenotype in Arabidopsis (de Oliveira et al. 2019), which may be due to incomplete complementation of the *atppa-at* mutant by *BIGE6* in vegetative tissues (Fig. 6E).

By contrast, we failed to detect rescue of *atppa-at* by a transgene expressing cytosolic PPA-AT (*AtPPA-AT^pro^:AtPPA-AT-C* and *AtPPA-AT^pro^:BigE6Like*) in any of 5 transformation experiments. We screened transformants from 4 independent transformations of the *atppa-at-2* mutant allele and 1 transformation of the other null allele, *atppa-at*-*1*. These results indicated that at a minimum the cytosolic PPA-AT is not sufficient to rescue female gametophyte function in the *ppa-at* mutant plants. This contrasts with the maize *bige6* mutant that only carries cytosolic BIGE6LIKE but can still support plant growth and development (Fig. 2). Normal 3:1 Mendelian segregation of *bige6* (WT:*bige6* = 218:72, *P* value = 0.95) indicates that *BigE6* is not required for female gametophyte function in maize.

### Plants lacking plastidial PPA-AT maintain overall homeostasis for Phe and Tyr and downstream metabolites in *bige6* leaves

**Figure 7.**
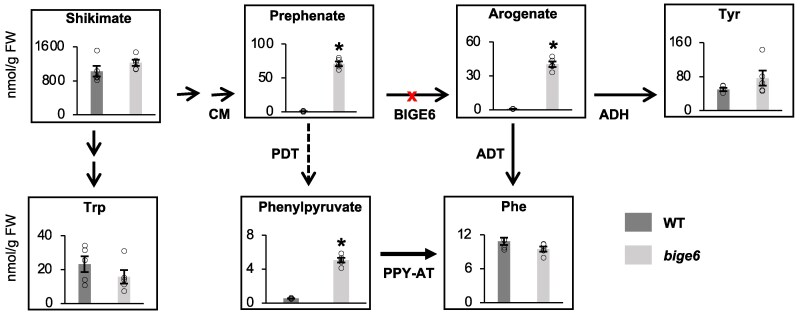
Metabolite profiles of *bige6* leaves reveal homeostasis of aromatic amino acid biosynthesis. Free aromatic amino acids and intermediates in aromatic amino acid biosynthetic pathways were measured in 10 DPG leaves. Five biological replicates were used for quantitation of the metabolites. Quantitated values of indicated metabolites in each biological sample are shown with white circles. Mean values ± SE are reported as nmol/g per fresh weight (FW). * indicates significant differences between WT and *bige6* with *P* < 0.05 by Student's *t* test.

In planta, PPA-AT function has been difficult to assess due to the lethality of Arabidopsis *ppa-at* mutant (Pagnussat et al. 2005) (Fig. 6). The viability of the maize *bige6* mutant plants (Fig. 2) now allowed us to examine the function of plastidial PPA-AT in biosynthesis of Phe and Tyr and their derived downstream metabolites in planta. For this purpose, *bige6* mutant and WT seedling leaves were sampled at 10 days postgermination (DPG) and the levels of various metabolites were analyzed. Total free Phe, Tyr, and Trp levels in *bige6* did not differ significantly from WT on a fresh weight basis (Fig. 7), indicating that homeostasis of aromatic amino acid levels was maintained in *bige6* leaves. By contrast, metabolic intermediates in the arogenate pathway showed marked differences between WT and *bige6* (Fig. 7). Prephenate levels were increased by about 70-fold in the mutant consistent with the expected block in conversion of prephenate to arogenate catalyzed by PPA-AT or BIGE6. Surprisingly, however, the PPA-AT product, arogenate, also accumulated in the mutant to a level 40-fold greater than in WT. This result suggests that the other PPA-AT, BIGE6LIKE, potentially provided the alternative route to synthesize arogenate. Phenylpyruvate was also increased by 10-fold in *bige6* mutant leaves, consistent with the presence of a cytosolic phenylpyruvate pathway.

**Figure 8.**
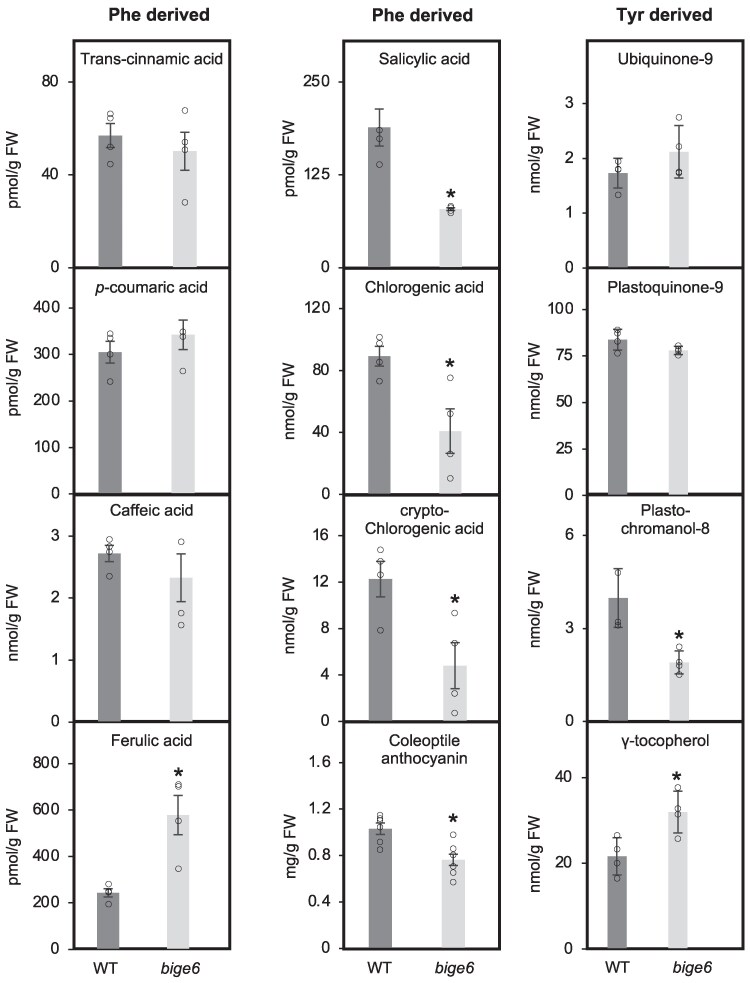
Metabolite profiles of *bige6* leaves reveal resilience in accumulation of specialized metabolites derived from Phe and Tyr. At least 4 biological replicates were used for quantitation of the metabolites. Quantitated values of indicated metabolites in each biological sample are shown with white circles. WT, wild type. FW, fresh weight. Data are means ± SE. * means differ at P < 0.05 based on Student's *t* test.

Consistent with the unchanged Phe level (Fig. 7), trans-cinnamic acid, which is produced by phenylalanine ammonia lyase from Phe, remained unaltered in *bige6* leaves. Levels of *P*-coumaric acid and caffeic acid were also comparable between WT and *bige6* mutant, whereas ferulic acid was 3 times higher in the mutant than in WT (Fig. 8). Of phenylpropanoid compounds measured only cryptochlorogenic, chlorogenic acid and coleoptile anthocyanins were decreased in the mutant. The latter result contrasts with the increased anthocyanin accumulation observed in *bige6* aleurone (Fig. 2F). Therefore, the impact of *bige6* on anthocyanin biosynthesis depends on developmental context. While it is currently unclear whether Phe is a precursor of salicylic acid in maize, we observed a marked decrease in total salicylic acid content of *bige6* leaves (Fig. 8). Analysis of various quinones derived from Tyr showed that with 2 exceptions these compounds were not measurably altered in the *bige6* mutant on a fresh weight basis. The exceptions were an increase in γ-tocopherol and a decrease in plastochromanol-8, which is formed by cyclization of plastoquinol-9 (Fig. 8). On a molar basis, the increase in γ-tocopherol was 4-fold greater than the decline in plastochromanol-8, resulting in an unexpected net increase in tocochromanol content of *bige6* leaves (Fig. 8).

Together, these data indicate that overall homeostasis is maintained in key pathways downstream of aromatic amino acid biosynthesis in *bige6*, which is consistent with unaltered levels of aromatic amino acid pool sizes and highlights resilient regulation of metabolism in relation to growth rate.

### The loss of plastidial PPA-AT perturbs amino acid homeostasis in *bige6* embryo and endosperm

**Figure 9.**
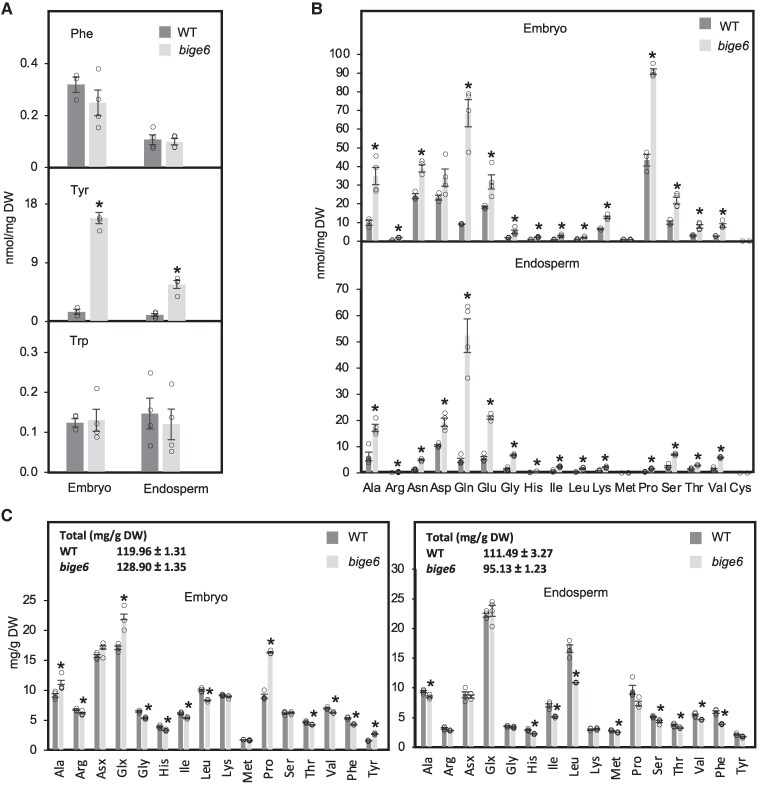
The *bige6* mutant perturbs amino acid homeostasis in the embryo and endosperm. Amino acid profiles were analyzed in mutant and WT embryos and endosperms harvested from self-pollinated ears segregating *bige6* at 24 DAP. DW, dry weight. **A)** Comparison of free aromatic amino acid levels in embryo and endosperm between WT and *bige6*. Comparison of free amino acids (**B**) and PBAAs (**C**) in embryo and endosperm between WT and *bige6*. Four biological replicates were used for these analyses. Quantitated values of indicated metabolites in each biological sample are shown with white circles. Data are means ± SE. *P < 0.05, Student's *t* test.

While *bige6* plants lacking plastidial PPA-AT evidently maintain overall homeostasis by reducing plant growth rate, *bige6* seeds grow to altered composition particularly in endosperm where accumulation of zein storage proteins is reduced (Fig. 2). To determine how the loss of the plastidial PPA-AT (i.e. BIGE6) impacts amino acid metabolism and protein synthesis in developing maize kernels, we quantified free amino acids (FAAs) as well as protein-bound amino acids (PBAAs) in *bige6* and WT (Fig. 9). Developing embryos and endosperms of WT and *bige6* mutant kernels were sampled from self-pollinated heterozygous plants at a late grain filling stage (24 DAP). Overall, total FAA contents were increased around 2.4-fold in the *bige6* mutant embryo and 4-fold in the mutant endosperm (Fig. 9, A and B). While free Phe and Trp levels were not altered in either embryo or endosperm of *bige6*, free Tyr levels were sharply elevated in both seed organs (Fig. 9A), which is in contrast to at most minor alteration in free Tyr levels in *bige6* leaves (Fig. 7). At least 15 of 17 FAAs quantified were elevated in *bige6* seed organs with differential accumulation of 7 abundant amino acids: Gln, Glu, Asn, Asp, Ala, Pro, and Ser, accounting 79.7% of the increase (Fig. 9B). In most cases, FAA increases were qualitatively similar in embryo and endosperm. Notable exceptions include Pro and Lys which showed substantially larger absolute increases in embryo than in endosperm (Fig. 9B). In contrast to the mostly parallel effects of *bige6* on FAA contents of embryo and endosperm, *bige6* differentially affected PBAA profiles of the filial organs of the seed. Total protein increased in *bige6* embryo and decreased in endosperm relative to WT (Fig. 9C). In embryo, differentially accumulated protein was enriched in Pro, Ala, and Glu/Gln, whereas in mutant endosperm protein showed relative depletion in Phe, Leu, and Ile. In contrast to the *bige6* effect on protein-bound Phe, the Tyr content of endosperm protein was not affected (Fig. 9C). These data indicate that *bige6* has major impacts on amino acid and protein composition of embryo and endosperm, in both similar and distinct manners.

### Loss of plastidial PPA-AT reprograms transcriptomes of *bige6* embryo and endosperm

To determine the molecular basis for *bige6* enhancement of embryo size, we performed RNA-seq analysis of developing embryo and endosperm sampled at 20 DAP by using a 1.5-fold change cutoff for genes with a minimum fragments per kilobase million (FPKM) value of 2. While comparable numbers of differentially expressed genes (DEGs) were detected in embryo and endosperm of the *bige6* mutant (2,495 vs. 3,178), the balance between upregulated and downregulated DEGs differed markedly in the 2 organs. In embryo, numbers of up- and downregulated DEGs were nearly equal (1,275 vs. 1,220 DEGs, respectively), whereas in endosperm upregulated DEGs outnumbered downregulated DEGs by >6-fold (2,734 vs. 444) (Supplementary Data Set 1). This striking contrast highlights qualitative differences in negative regulation of genes to shaping the response of embryo and endosperm to perturbation of aromatic amino acid biosynthesis.

Consistent with the opaque phenotype and reduced abundance of zein proteins in *bige6* endosperm (Fig. 2, C and G), at least 18 of the 444 DEGs downregulated in *bige6* endosperm encode zein proteins. The association of reduced zein accumulation with sharply lower steady-state zein mRNA abundance suggests 2 possibilities: (i) *bige6* perturbation of metabolism leads to negative transcriptional regulation of zein genes and/or (ii) translation of zein mRNAs is inhibited by starvation for aromatic amino acids, and the inefficiently translated mRNAs are degraded at a faster rate (Schmidt et al. 1992; Landry et al. 2002; Hartings et al. 2011; Zhang et al. 2015; Zhang et al. 2016b).

Among the upregulated DEGs in *bige6* endosperm, at least 353 genes (12.9%) have functions related to protein translation. Of those, 277 genes (10.1%) encode cytosolic ribosomal proteins. An enrichment of translation-related proteins and ribosomal protein genes also stood out in the upregulated DEGs in *bige6* embryo (15.1% and 13.1%, respectively). Enrichment of ribosome biosynthesis and protein translation functions among upregulated DEGs in *bige6* embryo and endosperm was confirmed by analyses of GO terms (Table 1). In addition, a large number of genes in upregulated class in both embryo and endosperm are related to RNA biogenesis, suggesting that posttranscriptional regulation plays an active role in the seed response to the loss of plastidial PPA-AT. While growth-related processes of ribosome assembly and protein translation were strongly upregulated in *bige6* seed organs, autophagy genes were downregulated in both embryo and endosperm. Together, these dramatic transcriptome shifts are broadly consistent with amino acid signaling and regulation, suggesting that *bige6* perturbation of amino acid homeostasis is a basis for stimulation of embryo growth.

**Table 1.** Functions enriched in *bige6* DEGs^a^

Genes upregulated in embryo and endosperm	Total	DEGs	*P*-value
Translation	712	120	0
rRNA processing	147	27	0
Ribosome biogenesis	83	20	0
Ribosomal large subunit biogenesis	38	15	0
U4 snRNA 3′-end processing	24	11	3.3E−13
Nuclear-transcribed mRNA catabolic process, Exonucleolytic, 3′–5′	25	11	5.3E−13
Nuclear mRNA surveillance	17	9	6.4E−12
Ribosomal small subunit assembly	45	12	3.0E−11
Nuclear polyadenylation-dependent mRNA catabolic process	14	8	3.4E−11
U1 snRNA 3′-end processing	15	8	6.2E−11
U5 snRNA 3′-end processing	15	8	6.2E−11
Nuclear polyadenylation-dependent rRNA catabolic process	32	10	1.6E−10
Nuclear polyadenylation-dependent tRNA catabolic process	32	10	1.6E−10
rRNA metabolic process	28	9	7.7E−10
Protein import into mitochondrial matrix	36	9	8.2E−09
Exonucleolytic trimming to generate mature 3′-end of 5.8S rRNA	36	8	1.1E−07
Exonucleolytic catabolism of deadenylated mRNA	62	10	1.3E−07
maturation of LSU-rRNA	28	7	2.1E−07
Ribosomal small subunit biogenesis	25	6	1.4E−06
Ribosomal large subunit assembly	39	7	2.4E−06
Maturation of SSU-rRNA	55	8	3.5E−06
ncRNA processing	19	5	4.0E−06
Cytoplasmic translational elongation	11	4	4.5E−06
**Genes upregulated in endosperm only**			
Translation	712	115	0
rRNA processing	147	33	2.2E−10
Protein folding	299	48	3.1E−09
Ribosome biogenesis	83	21	3.2E−08
Ribosomal large subunit assembly	39	13	3.0E−07
Maturation of LSU-rRNA	28	11	3.4E−07
rRNA metabolic process	28	11	3.4E−07
Box C/D snoRNP assembly	11	7	6.6E−07
Mitochondrial translation	20	9	8.1E−07
Formation of cytoplasmic translation initiation complex	28	10	2.3E−06
Chaperone-mediated protein complex assembly	10	6	4.2E−06
mRNA processing	198	30	5.6E−06
DNA replication	124	22	6.5E−06
**Genes upregulated in embryo only**			
Positive regulation of response to water deprivation	51	8	4.7E−07
**Genes downregulated in endosperm**			
Molecular function: nutrient reservoir activity	64	15	0
Starch biosynthetic process	42	6	2.1E−06
Cellular compartment: autophagosome	17	4	4.1E−06
**Genes downregulated in embryo**			
No enrichments for terms above the 10 gene threshold.			

^a^Enrichments of GO terms (biological process category unless specified) that are shared by least 10 maize genes (Gramene.org) were calculated using a binomial statistic. Enrichments with probabilities < 1.0E−05 are shown.

### The *bige6* mutant alters expression of key amino acid biosynthetic and catabolic pathways in embryo and endosperm

**Figure 10.**
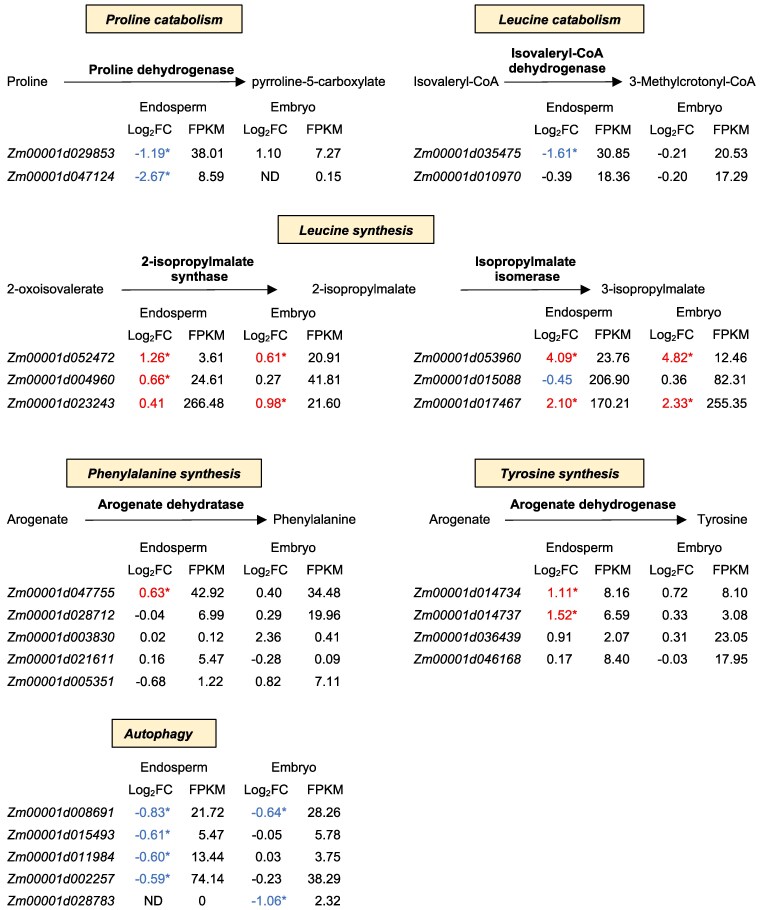
The *bige6* mutant alters expression of genes in key amino acid biosynthetic and catabolic pathways in embryo and endosperm. DEGs with functions in amino acid biosynthesis and catabolism pathways were identified (FDR 0.05) in transcriptomes of *bige6* and WT embryo and endosperm sampled at 20 DAP with 3 biological replicates from segregating ears of self-pollinated heterozygotes. To enable comparison of the relative contributions of gene family members, log_2_ of fold change (Log_2_FC) values of DEGs (red, upregulated; blue, downregulated) and paralogs that encode the same enzyme are shown together with FPKM values of WT. DEGs with >1.5-fold change are marked with asterisks. ND, not determined because the divisor is 0.

Our transcriptome analysis also gave insight into the molecular basis for altered amino acid metabolism in *bige6* seed. In *bige6* endosperm, changes in FAA levels described above were associated with altered expression of corresponding amino acid catabolic and biosynthesis pathway genes (Fig. 10 and Table 1). Consistent with this response, Pro accumulation in *bige6* endosperm was associated with downregulation of 2 proline dehydrogenase genes (*Zm00001d029853* and *Zm00001d047124*). Similarly, increases in free Leu and Ile in endosperm were associated with differential expression of branched-chain amino acid catabolic and biosynthetic pathway genes. One of 2 maize *isovaleryl-CoA dehydrogenase* (*IVD*) paralogs (*Zm00001d035475*) encoding a key enzyme in Leu catabolism was downregulated in *bige6* endosperm. In Arabidopsis, the orthologous *ivd* mutant causes accumulation of 15 FAAs in seeds (Gu et al. 2010). Plausibly, downregulation of *Zm00001d035475* would contribute to an analogous physiological state in maize endosperm where 15 out of 17 FAAs measured accumulate in *bige6* endosperm (Fig. 9B). Conversely, key genes in the Leu biosynthetic pathway were upregulated in *bige6* endosperm and embryo. These include the sole gene encoding the small subunit of isopropylmalate isomerase (*Zm00001d017467*), one of 2 large subunit genes (*Zm00001d053960*), and 2 of 3 endosperm-expressed 2-isopropylmalate synthase genes (*Zm00001d052472* and *Zm00001d004960*). In addition, several genes that act downstream of *BigE6* in aromatic amino acid biosynthesis were upregulated in *bige6* endosperm consistent with a transcriptional response to aromatic amino acid deficiency. These included 2 of 4 ADH paralogs for Tyr biosynthesis, *AroDH1* (*Zm00001d014734*) and *AroDH2* (*Zm00001d014737*), and an ADT gene for conversion of arogenate to Phe (*Zm00001d047755*). By contrast, differential regulation of shikimate kinase genes acting upstream of BIGE6 in synthesis of chorismate included a combination of upregulated (*Zm00001d052247*, *Zm00001d030688,* and *Zm00001d018061*) and downregulated paralogs (*Zm00001d002128* and *Zm00001d030109*). With the exception of Leu biosynthetic pathway genes (Fig. 10), the *bige6* embryo transcriptome overall included fewer changes in amino acid biosynthetic genes. Moreover, in contrast to endosperm, proline dehydrogenases were upregulated in *bige6* embryo. Together, these data reveal gene expression changes that likely contribute to the broad perturbation of amino acid homeostasis observed in *bige6* seed.

## Discussion

Our results shed light on roles of aromatic amino acid biosynthesis in seed and plant development. Loss of function of the plastid-localized PPA-AT, a key enzyme in the arogenate pathway for Phe and Tyr biosynthesis, has unexpected metabolic and developmental impacts in maize. We suggest that these phenotypes are due in part to presence of a cytosol-localized PPA-AT enzyme encoded by the *BigE6* paralog, *BigE6Like*. A capacity for arogenate synthesis in cytosol appears to be common, if not universally conserved, in flowering plants. In Arabidopsis, the single *PPA-AT* gene encodes both plastidial and cytosolic forms of the enzyme through alternative splicing. Although the fate and developmental roles of cytosolic arogenate are not yet fully understood, the cytosolic pathways for Phe and Tyr synthesis are evidently sufficient for seed formation and production of fertile plants at least in maize. By contrast, the cytosolic PPA-AT alone does not support female gametophyte development in Arabidopsis. In maize leaves, *bige6* plants lacking plastidial PPA-AT maintain overall homeostasis of aromatic amino acids as well as downstream products in response to a sharp reduction in capacity for Phe and Tyr biosynthesis. By contrast, we attribute the counterintuitive large embryo phenotype of *bige6* to broad shifts in amino acid homeostasis in the seed that promote upregulation of growth capacity in both embryo and endosperm. An underlying basis for the shift in amino acid metabolism is partly revealed by changes in expression of key amino acid biosynthetic and catabolic genes particularly in endosperm. Hence, the underlying metabolic mechanisms that scale growth of plant and seed organs to attenuated Phe and Tyr biosynthesis are resilient but also conditioned by developmental context.

### *Bige6* uncovers evidence of a cytosolic arogenate pathway for synthesis of Phe and Tyr

The alternative pathways for synthesis of Phe and Tyr known in plants utilize arogenate and phenylpyruvate intermediates, respectively. Genetic evidence indicates that the arogenate pathway localized in plastids is the predominant source of Phe (Maeda et al. 2010; Maeda et al. 2011; Corea et al. 2012). In addition, a cytosolic phenylpyruvate and 4-hydroxyphenylpyruvate pathway for Phe and Tyr has been confirmed in several species (Schenck et al. 2017; Qian et al. 2019; Schenck et al. 2020) (Fig. 1). Consistent with this capacity, most vascular plants contain a cytosolic CM2 isoform that enables prephenate production in the cytosol (Qian et al. 2019). In maize, the *CM2* gene (*Zm00001d015509*) and *BigE6Like* encoding cytosolic PPA-AT are widely coexpressed (https://www.maizegdb.org/) (Supplementary Fig. S4), suggesting that a potential for synthesis of arogenate from prephenate in the cytosol exists in diverse tissues. Similarly, we have confirmed the expression of a cytosolic PPA-AT encoded by alternatively spliced transcripts from the single *PPA-AT* gene of Arabidopsis. The presence of PPA-AT in the cytosol is further supported by Arabidopsis proteome data (Ito et al. 2011). Thus, our study uncovers and provides evidence for the presence of the cytosolic, arogenate pathway of Phe and Tyr biosynthesis (Fig. 1).

Presence of cytosolic PPA-AT in maize and Arabidopsis suggests that a capacity for synthesis of arogenate in cytosol may be broadly conserved in monocots and dicots. The number of *PPA-AT* genes in plant genomes varies potentially giving rise to multiple mechanisms for dual targeting of the enzyme. Like Arabidopsis, the rice genome contains a single *PPA-AT* gene (*LOC_Os01g65090*). The *O. sativa* v7.0 annotation includes 2 *LOC_Os01g65090* transcripts, one of which encodes a predicted cytosolic protein by alternative splicing (Supplementary Data Set 2), similar to the Arabidopsis *PPA-AT* (Fig. 5). Transcript annotations of *Sorghum bicolor PPA-AT* (*Sobic*.*003G370800*) indicate potential for 3 protein isoforms via alternative splicing or transcription start site use, 2 of which are predicted to be cytosolic. Thus, alternative splicing and utilization of alternative transcription start sites of *PPA-AT* genes are likely common mechanisms for generating cytosolic PPA-AT in monocots as well as eudicots. Interestingly, in teosinte, ancestor of modern maize, *BigE6* and *BigE6Like* orthologs are predicted to encode plastid- and cytosol-targeted proteins as in maize (Supplementary Data Set 2). This suggests that the 2 differentially targeted forms of PPA-AT evolved prior to separation of maize and teosinte.

The implication that cytosolic PPA-AT partially compensates loss of the plastidial PPA-AT in maize raises the question of whether cytosolic ADT and/or ADH activities for conversion of arogenate to Phe and/or Tyr are also present. Evidence of transcript variants for cytosolic AtPPA-AT (Fig. 5) combined with prior identification of cytosolic Arabidopsis ADT enzymes (Rippert et al. 2009; Para et al. 2016; Bross et al. 2017; Qian et al. 2019) suggests a potential for a complete cytosolic arogenate pathway for Phe biosynthesis in Arabidopsis. ADT6, which has both ADT and PDT activities (Cho et al. 2007), encodes plastid and cytosol protein isoforms by utilizing alternative transcription start sites (Rippert et al. 2009; Bross et al. 2017; Qian et al. 2019). Although genetic evidence implicates cytosolic ADT6 in the phenylpyruvate pathway (Qian et al. 2019), the catalytic efficiency of ADT6 utilization of arogenate is around 100-fold higher than for prephenate (Cho et al. 2007). Hence, ADT6 conversion of cytosolic arogenate to Phe is plausible. Moreover, the monofunctional, arogenate-specific ADT3 enzyme is localized in cytosol (Para et al. 2016) as well as in plastids (Bross et al. 2017). Taken together, these findings are consistent with Arabidopsis having a complete cytosolic arogenate pathway at least for Phe biosynthesis. We further analyzed transcripts of maize *ADT* and *ADH* (*AroDH* in maize) genes to evaluate potential for expression of cytosol-localized isoforms of either enzyme. The maize genome has 7 *ADT* and 4 *AroDH* genes. Careful annotation of gene sequences has thus far revealed no evidence that any of the 4 *AroDH* genes encode a cytosol-localized protein. Six out of 7 ADTs include predicted N-terminal TPs and have ATG codons downstream of the predicted TP which can potentially encode cytosolic forms of the enzyme by the use of alternative transcription start sites, as reported in petunia *ADT3* and Arabidopsis *ADT6* (Qian et al. 2019). Annotations of 1 maize *ADT* (*Zm00001d047755*) indicate that alternative splicing at the 1st intron potentially generates transcript variants encoding 2 distinct protein coding sequences, one predicted to be localized in plastids and the other in the cytosol. Thus, there is support for a complete cytosolic arogenate pathway for biosynthesis of Phe in maize as well as in Arabidopsis although the distribution of this capacity within the plant is unknown.

### PPA-AT mutants uncover roles of plastidial and cytosolic arogenate pathways

The roles and relative contributions of plastidial and cytosolic pathways for Phe and Tyr biosynthesis in seed and plant development are poorly understood. Our analyses of *atppa-at* deletion alleles created by *CRISPR/Cas9* in Arabidopsis show that PPA-AT is indispensable for seed formation. More specifically, the plastid-localized PPA-AT is essential for female gametophyte development, which cannot be compensated by the cytosolic variant of transgene (Fig. 6). It is noteworthy that Arabidopsis *adt2* has a similar phenotype with reduced transmission likely through the female gametophyte, as implicated by many empty spaces in the self-fertilized siliques of the heterozygous mutant plants (El-Azaz et al. 2018). Hence, even though the embedded female gametophyte is in intimate contact with maternal tissues, transfer of Phe and Tyr from the sporophyte, if it occurs, is insufficient to rescue the female gametophyte in *atppa-at*. By contrast, we find no evidence of an analogous role of the plastidial PPA-AT (BIGE6) in the female gametophyte of maize. Rescued *atppa-at-2* plants that express only the plastidial PPA-AT develop normally indicating the cytosolic isoform does not have an essential developmental function. Consistent with this result, a *cm2* knockout, which is expected to inhibit both arogenate and phenylpyruvate pathways in cytosol, is viable and capable of maintaining normal Phe levels (Qian et al. 2019). Since Arabidopsis *CM2* expression is increased by wounding, Qian et al. (2019) have suggested that the cytosolic phenylpyruvate pathway is involved in stress responses. In that case, it is plausible that cytosolic arogenate synthesis also has a role in stress.

The viability of maize *bige6* indicates that one or both cytosolic pathways support seed formation and plant growth albeit at a sharply reduced rate. The sharply reduced growth rate of *bige6* plants (Fig. 2H) indicates that growth is likely limited by the rate of aromatic amino acid synthesis. Based on the reduction in total biomass of *bige6* plants (Fig. 2, H and J), the cytosolic pathways may contribute as much as 30% of the total aromatic amino acid synthetic capacity. We speculate that the unexpected accumulation of arogenate in *bige6* leaves is due to arogenate synthesis in the cytosol by BIGE6LIKE (Figs. 3 and 4). However, the apparent size of this pool in the mutant suggests that downstream conversion of cytosolic arogenate to aromatic amino acids is limiting. Plausibly, if cytosolic ADT and/or AroDH activities are low or absent, arogenate may instead be transported into the plastid to produce Phe and Tyr. Similarly, while detection of phenylpyruvate in *bige6* leaves is consistent with the conversion of prephenate to phenylpyruvate by PDT in cytosol (Maeda et al. 2011), the elevated phenylpyruvate accumulation (Fig. 7) suggests that transamination of phenylpyruvate to Phe may also be slow. Interestingly, in petunia, due to coupling of Trp-dependent transamination of phenylpyruvate to auxin synthesis, engineering increased flux through the phenylpyruvate pathway gives rise to a detrimental developmental phenotype (Lynch et al. 2020). By contrast, *bige6* plants do not have obvious auxin phenotypes, suggesting that Phe synthesis via phenylpyruvate may be limited in *bige6*.

Given these considerations overall, it is unclear whether or how an optimum balance between Phe and Tyr synthesis is attained in *bige6*. In *bige6* seed, free Tyr levels are higher than in WT (Fig. 9), suggesting that Tyr is in excess relative to Phe. In vegetative tissues, utilization of Phe likely exceeds demand for Tyr due to the synthesis of lignin and other secondary metabolites. While free Tyr is not significantly elevated in *bige6* leaves (Fig. 7), mutant leaves have a greater total accumulation of tocochromanols compared with WT (Fig. 8) consistent with a relative excess in available Tyr.

### *bige6* implicates amino acid signaling in regulation of embryo growth

Our analysis of *bige6* highlights a role for amino acid metabolism in regulation of embryo growth. Paradoxically, attenuation of aromatic amino acid biosynthesis in *bige6* promotes rather than retards embryo growth in contrast to small stature of plants in the mutants of aromatic amino acid biosynthesis including *bige6* itself (de Oliveira et al. 2019; Ren et al. 2024) (Fig. 2). This phenotype contrasts with embryo lethality and reduced fertility phenotypes conditioned by Phe- and Tyr-deficient *adt2* and *tyra2* mutants of Arabidopsis, respectively (El-Azaz et al. 2018; de Oliveira et al. 2019). We propose that the counterintuitive *bige6* embryo size phenotype results from a broad perturbation of amino acid homeostasis in embryo and endosperm that signals upregulation of growth processes (Fig. 9).

RNA-seq analyses of *bige6* embryo and endosperm reveal that transcriptomes of both organs are poised for increased growth relative to WT. Quantified in terms of number of genes involved, the scale of the transcriptome shift toward growth processes is substantially larger in endosperm than in embryo. This is partly attributed to upregulation of endosperm-specific genes for many ribosome and protein synthesis functions (Table 1). Nevertheless, only embryo growth is increased, whereas endosperm size is not measurably affected in *bige6* seed. Consistent with enhanced growth of the embryo, overall amino acid incorporation into protein is increased in *bige6* embryo relative to WT, whereas PBAAs are either unchanged or reduced in *bige6* endosperm (Fig. 9). A similar, apparently larger enhancement of embryo protein synthesis occurs in the Tyr-deficient *arodh1* mutant of maize (Holding et al. 2010). While *arodh1* endosperm has an opaque phenotype as in *bige6*, *arodh1* does not have a discernible embryo size phenotype. Whether this is due to redundancy of the *AroDH* gene family or indicative of a specific role for Phe in embryo size remains to be determined. Our results are consistent with the possibility that embryo and endosperm differ in their capacities for rebalancing of protein biosynthesis in response to changes in amino acid resource availability. In endosperm, which is rich in zeins, a decrease in zein accumulation in the mutant endosperm could restrict endosperm expansion, whereas in mutant embryos capacity for rebalancing of amino acids into diverse proteins might enable greater plasticity for growth.

Together, our metabolite and transcriptome analyses build a strong, albeit thus far circumstantial case that amino acid signaling accounts for the *bige6* embryo size phenotype. In diverse organisms, the balance between amino acid availability and growth capacity is regulated by the universally conserved TOR signaling complex (Shi et al. 2018; Takahara et al. 2020; Liu and Xiong 2022; Lutt and Brunkard 2022). The amino acids that exhibit large changes in steady-state concentration in *bige6* embryos include Gln, Arg, and Leu, which are known to be sensed by mTOR in other systems (Takahara et al. 2020). Moreover, a recent study implicates aromatic amino acid sensing in TOR signaling (Livneh et al. 2023). While mechanisms of amino acid sensing upstream of TOR in plants are less well understood, the functional categories of genes up- and downregulated in *bige6* mutant embryo and endosperm are broadly consistent with activation of TOR (Loewith and Hall 2011). Transcriptomes of *bige6* embryo and endosperm both reveal upregulation of ribosomal protein genes as well as genes for ribosome synthesis and assembly, mRNA processing, and protein translation (Table 1). In addition to the shared DEGs enriched in these categories, endosperm-specific upregulated DEGs are similarly enriched for roles in translation and ribosome assembly. In addition to promoting growth capacity, TOR negatively regulates autophagy (Mugume et al. 2020). Consistent with that response, key regulators of autophagy in maize (Li et al. 2015) are downregulated in both organs (Fig. 10).

### *bige6* alters regulation of key amino acid biosynthesis and catabolism pathways

The amino acid signaling hypothesis raises the question of how broad shifts in amino acid homeostasis arise from attenuation of Phe and Tyr synthesis in the seed. In this respect, our data reveal a dichotomy between embryo and endosperm (Fig. 10). While Leu biosynthetic genes are upregulated in both embryo and endosperm of *bige6* seed, Pro and branched-chain amino acid catabolic pathways are differentially expressed in the filial organs. In *bige6* endosperm, a modest elevation in free Pro is associated with downregulation of proline dehydrogenase genes involved in Pro catabolism. By contrast, the same gene is upregulated in *bige6* embryo, which has a comparatively large increase in Pro. In Arabidopsis, proline dehydrogenase is induced by excess Pro (O’Leary et al. 2020), suggesting that upregulation of Pro catabolism in the *bige6* embryo is a response to abundant Pro, whereas in endosperm downregulation of Pro catabolism likely contributes to net accumulation of Pro. Downregulation of branched-chain amino acid catabolic genes is detected only in endosperm. Interestingly, in Arabidopsis seed disruption of branched-chain catabolism causes a broad perturbation of amino acid homeostasis (Gu et al. 2010; Peng et al. 2015). Hence, downregulation of *IVD* in endosperm may impact levels of diverse amino acids. While amino acid levels are also broadly elevated in *bige6* embryo, altered expression of *IVD* is not apparent. Plausibly excess amino acids that accumulate initially in the endosperm are exported to the embryo.

As noted above, the unexpected increase in free Tyr in *bige6* embryo and endosperm suggests that Tyr is in excess relative to Phe. Nevertheless, the source of excess Tyr in *bige6* is unclear. We cannot rule out the possibility that the seed imports substantial amounts of aromatic amino acids from maternal tissues. The hypothesis that attenuated capacity for Phe and Tyr synthesis is partially ameliorated by maternal sources could account for the unexpected accumulation of anthocyanins derived from Phe in aleurone cells of *bige6* endosperm. Plausibly, close contact of aleurone with maternal pericarp tissues would facilitate uptake of Phe from the surrounding apoplast. The implication that maternal tissues are a substantial source of aromatic amino acids in the maize seed stands in stark contrast to the apparent absence of maternal aromatic amino acid transfer to the female gametophyte in Arabidopsis. There is growing evidence that pathways and regulation of aromatic amino acid biosynthesis have diversified substantially among plant species and may differ between tissues within a plant. In grasses, for instance, there are key differences in aromatic amino acid biosynthesis pathways that direct flux of Phe and Tyr to downstream metabolite synthesis (El-Azaz et al. 2023). Therefore, the diversity of mechanisms of aromatic amino acid biosynthesis and roles in plant development is worthy of investigation beyond model organisms.

## Materials and methods

### Plant growth conditions

Maize materials used in this study were grown at the Plant Science Research and Education experimental farm at Citra, FL, USA, in the spring and fall seasons. In winter, plants were grown in the greenhouse. For metabolite analyses, seeds were sown in the pots with soil in the chamber at 22 °C. The second leaf from seedlings was harvested for analysis at 10 DPG. Arabidopsis WT Col-0, transgenic, and mutant seeds were sterilized with 50% bleach for 5 min and rinsed with sterile water before being placed on Murashige and Skoog (MS) medium (1 × MS salt, 0.05% MES, 1% sucrose sterilized by filtration, and 0.07% of phytagel). After stratification at 4 °C for 2 d, plates were transferred to a plant growth room under a 16-h light and 8-h dark growth condition at 22 °C for 2 wk before being transferred to soil. *N. benthamiana* seeds were planted into the soil and grown under the same growth condition with *A. thaliana*.

### Mu-seq analysis of the *bige6* mutant

The *bige6-umu1* mutant was analyzed by Mu-seq. This method was performed as established (McCarty et al. 2013). Briefly, 6 nonsegregating and 6 segregating ears of *bige6-umu1* were pooled. Eight kernels from each ear were grown in soil and the genomic DNA was extracted from resulting seedlings. For Mu-seq library construction, genomic DNA was randomly sheared by sonication. After size selection, an adaptor U was ligated to the 3′ end. We first performed the first-round PCR to amplify DNA fragments using a Mu-specific primer TIR6 and a U adaptor primer. The following PCR was performed to introduce a Mu-seq primer at the 5′ end including the Illumina A1 sequencing adaptor, a 4-bp barcode, and the TIR region. An Illumina, A2-U sequencing adaptor was incorporated to the 3′ end. A third PCR completes the nesting of A-1 Illumina sequencing adaptor. Mu-seq library was sequenced using the Illumina NextSeq50.

### Phylogenetic analysis

For phylogenic tree construction, homologs of PPA-ATs were identified by using the Phytozome database (https://phytozome-next.jgi.doe.gov/). The amino acid sequences of PPA-ATs were aligned using MEGA11 by default settings (Tamura et al. 2021). The tree was constructed in MEGA 11 with a maximum likelihood method. Bootstrap was set as 1,000. Sequences used for the analysis are given in Supplementary Data Set 3.

### Histology

For embryo histological analysis, WT and *bige6* kernel were harvested at 28 DAP from the self-segregating heterozygous ear. Harvested kernels were first fixed in freshly prepared 4% formaldehyde solution for 24 h at 4 °C. The tissue was then dehydrated with an ethanol/histoclear series. Paraffin was used to replace the ethanol/histoclear mixture for embedding at 50 °C. Wax was changed twice a day for 2 d. Ten-micrometer sections were made with a microtome and stained in safranin O solution. For lignin staining, cross sections of the 8th internode of WT and *bige6-umu1* were stained with 2% phloroglucinol (w/v) for 5 min followed by 20% HCl for additional 5 min. Pictures of stained sections were captured using a Leica DMC4500 camera.

### Flow cytometry analysis

About 150 mg of 28 DAP embryos or 500 mg of endosperms was chopped in the 1.5 mL of ice-cold chopping buffer (20 mm MOPS, pH 7.2, 45 mm MgCl_2_, 30 mm Na_3_ citrate, 0.1% Triton X-100, and 0.01% RNase A) with a razor blade. The resulting homogenate was filtered with a 50-*μ*m nylon mesh and a 20-*μ*m nylon mesh separately. Samples were then centrifuged for 3 min at 94 *g* to pellet the nuclei. Pellet was resuspended in the chopping buffer with 0.05% propidium iodide and analyzed by the flow cytometer.

### Metabolite profiling

Zein protein analysis was performed using 28 DAP WT and *bige6-umu1* dry kernels as described (Holding et al. 2010). Whole kernels were weighted and ground into fine powder with liquid nitrogen. Borate extraction buffer (12.5 mm sodium borate, 1% SDS, 2% β-mercaptoethanol, pH 10) was then added proportionally to the flour (1 mL for 100 mg flour). The mixture was incubated at room temperature with shaking for 30 min. After centrifugation, 500 *μ*L of supernatant was removed to a new tube and mixed with 1.2 mL of ethanol. After overnight incubation, zein proteins were separated by centrifugation. 100 *μ*L of zein protein solution was dried in a vacuum concentrator and resuspended in the SDS loading buffer (120 mm Tris–HCl, pH 6.8, 2% SDS, 10% glycerol, 375 mm β-mercaptoethanol, and 0.025% bromophenol blue). A total of 10 *μ*L of zein solution was boiled and then loaded into a 12% SDS–PAGE gel. The gel was stained in the Coomassie blue R250. Oil content in mature dry kernels was predicted by skNIR spectroscopy analysis as described (Jiang et al. 2007; Gustin et al. 2013). To quantify starch, dry kernels were ground into fine powder by a mechanical homogenizer with metal beads, and the ground tissue was used for starch assay described in Boehlein et al. (2018).

Total anthocyanin was extracted from mature dried WT and *bige6-umu1* kernels or coleoptiles and quantified using the method described previously (Liu et al. 2018). Samples were weighed and homogenized in liquid N_2_. Total anthocyanin was extracted in 80% methanol solution containing 1% hydrochloric acid and incubated for 30 min. The supernatant was collected by centrifugation, which was then diluted with either 0.03 m potassium chloride buffer (pH 1.0) or 0.4 m sodium acetate buffer (pH 4.5). The absorbance of these 2 diluted mixtures was measured with a UV–Vis spectrophotometer at 520 and 700 nm, respectively. The anthocyanin content was quantified as follows: anthocyanin content (mg/g) = (A × MW × DF × 1,000×Vol)/(ɛ×1×W), where A is adjusted as (A_520_−A_700_)_pH1.0_−(A_520_−A_700_)_pH4.5_, MW is the molecular weight of cyandindin-3-glucoside (449.2), DF is the dilution factor, Vol is the volume of methanol solution, ɛ is the molar absorptivity of cyanindin-3-glucoside (26,900 cm^−1^ M^−1^), and W is the weight of samples.

An acetyl bromide method for soluble lignin quantification was used as described (Moreira-Vilar et al. 2014). The 8th internode of maize plants at flowering stage was ground to a fine homogeneous powder. The fine powder was washed with 70% ethanol, chloroform:methanol (1:1), and acetone separately for at least 3 times. After washing, samples were dried at 55 °C. Approximately 5 mg of dried residue was dissolved in 1 mL 25% (v/v) acetyl bromide, which was then diluted with glacial acid and incubated at 70 °C for 30 min. Samples were cooled on ice, and 5 mL of glacial acetic acid was added to the solution. A 300 *µ*L volume of the samples was diluted by adding 400 *µ*L 1.5 N sodium hydroxide and 300 *µ*L 0.5 m freshly made hydroxylamine hydrochloride. The absorbance of the mixture was measured at 280 nm with a spectrophotometer. An extinction coefficient of 17.75 g^−1^ L cm^−1^ for maize samples was used for lignin content calculation.

For ubiquinone analysis, second leaves (200–325 mg) were collected from 10 DPG maize plants, spiked with ubiquinone-10 as an internal standard (4.62 nmoles), and homogenized in 3 mL of 95% (vol/vol) ethanol using a 5-mL Pyrex tissue grinder. For plastoquinone-9, plastochromanol-8, and tocopherol analyses, extract aliquots were clarified by centrifugation (21,000 *g*; 5 min) and immediately analyzed by reverse-phase chromatography using diode array and fluorometric detections as previously described (Block et al. 2013). For ubiquinone analysis, 1 mL of extract was mixed to 0.5 mL of water and phase partitioned twice with 5 mL of 100% hexane. Hexane fractions were evaporated to dryness with gaseous N_2_, and samples were resuspended in 0.5 mL of 95% (vol/vol). Sample aliquots (0.2 mL) were oxidized by adding 4 mL of a 30% (vol/vol) solution of hydrogen peroxide, centrifuged at 21,000 *g* for 5 min, and then immediately analyzed by reverse-phase chromatography using diode array detection as previously described (Tang et al. 2022). Metabolites were quantified according to external calibration standards, and data were corrected for the recovery of ubiquinone-10.

For soluble phenylpropanoid metabolite and flavonoid analysis, second leaves were detached from 10 DPG WT and *bige6* seedlings and stored in liquid nitrogen immediately. Metabolites were quantified by liquid chromatography–tandem mass spectrometry (LC–MS/MS). To extract metabolites, 100 mg of ground tissue was mixed with 500 *μ*L of phytohormone extraction buffer (1-propanol/water/HCl [2:1:0.002 vol/vol/vol]) containing 500 nm  ^13^C_6_-benzoic acid (ICON Isotopes), d4-SA (Cayman Chemical), and d5-trans-cinnamic acid (CDN Isotopes), as well as 1.25 mm Zirmil Y beads. Samples were twice homogenized on a Precellys 24 homogenizer (Bertin Corp.) for 30 s at 6,000 *g*. The samples were then shaken for 30 min in darkness. Then, 500 *μ*L of dichloromethane was added to the samples and they were briefly vortexed before being centrifuged at 14,000× *g* for 5 min. The lower organic layer was moved to a glass vial and evaporated under a nitrogen gas stream. Samples were resuspended in 150 *μ*L of methanol and stored overnight at −20 °C. All samples were then syringe-filtered through 0.20-*μ*m polytetrafluoroethylene filters (Millipore, Burlington) and into inserts for analysis by LC–MS/MS. A Vanquish HPLC (Thermo) connected to a TSQ Quantis mass spectrometer (Thermo) was used for analysis. An injection volume of 3 *μ*L and a mobile-phase flow rate of 300 *μ*L min^−1^ were used, with the column temperature set to 25 °C. The mobile phases consisted of 0.2% acetic acid in water (Solution A) and 0.2% acetic acid in acetonitrile (Solution B). Separation of target analytes was performed on a Hypersil GOLD C_18_ column (Thermo) (150 × 2.1 mm; 1.9 *μ*m particle size) with a mobile-phase gradient of (time—%B): 0 min—0%, 13 min—50%, 13 min—100%, 16 min—100%, 22 min—0%, and —stop. Analytes were ionized by electrospray ionization (ESI) and detected via scheduled multiple reaction mentoring in negative mode. The voltage was −3000 v, the ion transfer tube temperature was 300 °C, and the vaporization temperature was 350 °C. The sheath, auxiliary, and sweep gases were set to 50, 15, and 2, respectively. Response factors based on internal and authentic external standards for all compounds and the concentrations of analytes were determined with the labeled internal standards and response factors.

For aromatic amino acids and shikimate pathway intermediate analysis in leaves, around 30–40 mg of pulverized fresh second leaves detached from 10 DPG maize seedlings was resuspended into 400 *µ*L of methanol:methanol (2:1), with 25 *µ*M of norvaline as internal standard, for ∼1 h in the fridge and vortexed. After centrifugation at 20,000 *g* for 5 min, the whole supernatant was transferred to a fresh tube and mixed with 300 *µ*L of 1% 2-amino-2-methyl-1-propanol buffer pH 10, followed by adding 125 *µ*L of chloroform, vortexed for 2 min, and spun down at 20,000 *g* for 5 min for phase separation. Around 500 *µ*L of the upper aqueous phase was then transferred to a fresh tube and dried for 4 h in a centrifugal vacuum concentrator at 40 °C. The resulting pellets were resuspended into 100 *µ*L of 80% methanol by vortexing and sonication in a water bath for 5 min, followed by centrifugation at 20,000 *g* for 5 min. Supernatants were subjected for injection using a Vanquish Horizon Binary UHPLC (Thermo Scientific) coupled to a Q Exactive mass spectrometer (Thermo Scientific). Two microliters of each sample were analyzed with a InfinityLab Poroshell 120 HILIC-Z column (150 × 2.1 mm, 2.7-μm particle size; Agilent) in a gradient of 5 mm ammonium acetate/0.2% acetic acid buffer in water (solvent A) and 5 mm ammonium acetate/0.2% acetic acid buffer in 95% acetonitrile (solvent B) at a flow rate of 0.45 mL/min and column temperature of 40 °C. The phase B gradient was: 0–2 min, 94%; 2–9 min, 94–88%; 9–19 min, 88–71%; 19–20 min, 71–20%, 20–21.5 min, 20%; 21.5–22 min, 20–94%; and 22–25 min, 94%. Compound abundance was calculated based on authentic standards and normalized by the recovery factor of the internal standard (norvaline).

Amino acid analysis was performed on WT and *bige6-umu1* endosperms and embryos sampled at 24 DAP as described in Yobi and Angelovici (2018) and Yobi et al. (2020). For the PBAA fraction, ∼3 mg of dry seeds (*n* = 4) was hydrolyzed with 6N HCl for 24 h at 110 °C. After hydrolysis and filtration, 10 *µ*L of the hydrolysate was transferred to clean tubes and evaporated to dryness. The pellets were then reconstituted with Milli-Q purified water containing 13 internal standards and analyzed using a Xevo TQ Absolute ultra-performance liquid chromatography–tandem mass spectrometer instrument (UPLC–MS/MS) (Waters Corporation) as described (Yobi and Angelovici 2018). During acid hydrolysis, tryptophan (Trp) and cysteine (Cys) were lost, asparagine (Asn) was converted to aspartic acid (Asp), and glutamine (Gln) was converted to glutamic acid (Glu); therefore, Asx represents a combination of Asn and Asp, and Glx represents a combination of Gln and Glu. This treatment also destroys all the FAAs, leaving only the PBAAs. For the FAA fraction, water extraction was used as detailed in Yobi et al. (2020). Briefly, ∼5 mg of seed tissue (*n* = 4) was extracted with Milli-Q purified water containing 13 internal standards and analyzed the same way as for the PBAA. For both fractions, serial dilutions of external standards were analyzed alongside the samples for accurate identification and quantification. Separation was done with a Kinetex LC column (2.6 *µ*m, C18, 100 Å, 100 × 21 mm; Phenomenex) maintained at 30 °C. The injection volume was set to 10 *µ*L, and the flow rate was set to 0.3 ml/min. The mobile phase A consisted of 1 mm of the ion-pairing agent perfluoroheptanoic acid, while acetonitrile served as the mobile phase B. The flow gradient was set as follows for B: 98% at 0 min; 80% at 0.1 min; 60% at 2.3–3.6 min; and 98% at 4.0–5.98 min. The MS ESI in positive mode and multiple reaction monitoring transition for each compound were used for acquisition of mass spectra. Flow gas and desolvation were set to 150 and 500 l/h, respectively. Desolvation temperature was set to 350 °C, and the collision gas flow was set to 0.15 mL/min. After run completion, the data were retrieved and analyzed using the MassLynx data analysis software (TargetLynx XS, Waters), exported to Excel sheets and back calculated to total volume and sample weight to obtain the final amounts in nmol/mg tissue.

### RNA extraction and RT-PCR

For RNA extraction, 2-wk-old maize seedling leaf and Arabidopsis WT Col-0 root were harvested. Total RNA was isolated from harvested tissues using a RNeasy plant mini kit (Qiagen) as the protocol described. Isolated RNA was treated with DNaseI to remove genomic DNA (Thermo Fisher Scientific). One microgram of trimmed total RNA was reverse-transcribed to cDNA with the Agilent cDNA reverse transcription kit (Agilent). Gene-specific primers were used to amplify fragments, and PCR products were confirmed by sequencing. Primers are listed in Supplementary Table S1.

### RNA-seq analysis

For RNA-seq analysis, WT and *bige6-umu1* mutant embryos and endosperms were dissected from three 20 DAP self-pollinated heterozygous ears harvested from the field (Citra, FL, USA), and total RNA was isolated from the pooled embryo and endosperm tissue. RNA-seq libraries were constructed using NEBNext Ultra RNA Library Prep Kit for Illumina (New England Biolabs) according to the manufacturer's protocol. Briefly, mRNA was first purified and fragmented followed by separate first-strand and double-strand cDNA synthesis. The end repair of cDNA was performed, and the adaptor for Illumina sequencing was then ligated to the end. The DNA libraries were enriched, and the quality was validated using an Agilent Technologies 2100 Bioanalyzer. Finally, the library was sequenced with an Illumina NextSeq500.

The regenerated reads were first evaluated by FastQC to trim low-quality reads. Then, the resulting reads were mapped to the maize genome (B73 RefGen_v4, AGPv4) using Tophat2. After mapping, Cufflinks was used to perform transcript assembly and calculate FPKM for genes. Cuffmerge and Cuffquant were used subsequently for merging together assemblies and computing gene expression levels. The DEGs among different samples were compared and identified by the Cuffdiff program. The RNA-seq data are uploaded to the NCBI SRA database (accession number: PRJNA368967).

### GO term analysis

GO term annotations for B73v5 genes were obtained from MaizeGDB (https://download.maizegdb.org/GeneFunction_and_Expression/Pannzer_GO_Terms). Enrichment for Molecular Function and Biological Process terms was evaluated by calculating a binomial probability for observed frequency in each class of upregulated and downregulated genes using the frequency of the GO term in the total set of annotated genes as the expected value. Enrichments were calculated for terms that occurred in at least 10 genes.

### Protein expression and PPA-AT enzyme assays

The cDNA coding sequences of *BigE6, bige6-umu2,* and *BigE6Like* were first cloned using gene-specific primers described in Supplementary Table S1 and ligated to Zero-Blunt TOPO vector (Thermo Fisher Scientific). To generate the His-tagged recombinant proteins, the open reading frames of *BigE6, bige6-umu2,* and *BigE6like* were amplified from the reconstructed TOPO template with gene-specific primers to introduce BamHI and XhoI digestion sites. The amplified products were then digested with BamHI and XhoI and subcloned into digested pET28b vector (Novagen). In each step of PCR amplification, sequencing was used to confirm no errors in the sequence. The recombinant pET28b vector was transformed into *E. coli* BL21 (DE3) cells (Thermo Fisher Scientific). For recombinant protein purification, *E. coli* cells transformed with the pET28a vectors carrying individual *PPA-AT* genes were incubated at 37 °C in LB medium containing 50 mg/mL kanamycin until the OD_600_ reached 0.5. The 0.4 mm isopropyl β-D-1-thiogalactopyranoside was added to induce protein expression at 16 °C overnight. The cells were pelleted by centrifugation at 4,000 *g* for 20 min and resuspended in the binding buffer (500 mm NaCl and 20 mm Tris–HCl, pH 8.0). After sonication, the supernatant and inclusion body were separated by centrifugation. Soluble supernatant fractions were passed through Ni-NTA His-bind columns (Bio Basic) and washed with 2 × column volume wash buffer (500 mm NaCl and 20 mm Tris–HCl, pH 8.0, 20 mm imidazole). Recombinant proteins were eluted with the buffer solution (500 mm NaCl and 20 mm Tris–HCl, pH 8.0, 250 mm imidazole). The expression of target proteins was confirmed by SDS–PAGE gel.

The aminotransferase reactions were initiated by adding the enzyme solution (final concentration; 20 *µ*g/µL) to the preheated 50 mm sodium phosphate reaction buffer containing 1 mm prephenate, 5 mm Asp or Glu, and 200 *µ*M pyridoxal-5′-phosphate (Maeda et al. 2011; Dornfeld et al. 2014). After incubation at 37 °C for an hour, the reactions were stopped by adding methanol (v/v 66%). Ten microliters of the resulting solution was injected into HPLC (Agilent 1260 equipped with an Eclipse Plus XDB-C_18_ column) to confirm arogenate production as o-phthaldialdehyde derivatives (Maeda et al. 2010). Arogenate, aspartate, and glutamate were separated at a flow rate of 0.5 mL/min with a 0–10 min 10–30%; 10–15 min 30–70%; 15–19 min 70–10%; and 19–23 min 10% methanol in 0.1% (v/v) ammonium acetate (pH 6.8) and detected at UV absorbance at 336 nm.

### CRISPR/Cas9 vector construction and mutant screening

The plasmid pHEE401E carrying a *Cas9* gene and the pCBC-DT1T2 site where 2 guide RNAs (gRNAs) nested were used for *CRISPR/Cas9* gene editing (Xing et al. 2014; Wang et al. 2015). For vector construction expressing 2 gRNAs, 2 primer pairs incorporated with 2 gRNA sites targeting *AtPPA-AT* (*At2G22250*) were used to amplify pCBC-DT1T2 for assembly of gRNAs to PCR fragments. A golden gate method was employed for introducing gRNAs to Cas9 vector. PCR fragment and pHEE401E were digested with BsaI and ligated with T4 ligase as described (Weber et al. 2011). The recombinant constructs were introduced into *Agrobacterium tumefaciens* GV3101 and then transformed into Col-0 line using the floral dip method as described (Clough and Bent 1998). For mutant screening, transgenic plants were selected on MS medium with hygromycin resistance. Genomic DNA was extracted from positive transgenic plants, and targeted region was PCR-amplified from DNA followed by sequencing for selecting mutants. Mutant lines were backcrossed with Col-0 to remove *Cas9* and nontargeted mutants. *Cas9*-free mutant lines were selected for experiments. PCR primers and sequencing primers are listed in Supplementary Table S1.

### *N. benthamiana* transient expression and protoplast isolation

To generate the GFP fusion protein, the coding regions (without a stop codon) of *BigE6*, *BigE6* with no TP, *BigE6Like*, *BigE6Like* with TPs, *AtPPA-AT,* and *AtPPA-AT* without TPs were amplified with gene-specific primers in Supplementary Table S1. PCR products were then subcloned into the gateway entry vector pENTR/TOPO vector (Thermo Fisher Scientific). After sequencing, genes then were subcloned in pK7FWG2 (C-terminal eGFP). The recombinant constructs were introduced into *A. tumefaciens* GV3101. For detecting protein subcellular localizations, the GFP fusion constructs were transiently expressed in *N. benthamiana* leaves as described with some modifications (Sparkes et al. 2006). Overnight *Agrobacterium* cell cultures carrying GFP fusion proteins (Lakatos et al. 2004) were transferred to 50 mL of YEB including 20 *µ*M acetosyringone and incubated at 28 °C until the cell density reached the OD_600_ of 0.5 to 0.8. The cells were then pelleted by centrifugation, washed, and resuspended in the infiltration medium containing 10 mm MES–KOH (pH 5.6), 10 mm MgCl_2_, and100 *μ*M acetosyringone to a final OD_600_ of 0.5. Cells were incubated for additional 2–3 h at room temperature and infiltrated to abaxial side of 6-wk-old *N. benthamiana* plant leaves. Two to five days after infiltration, protoplasts were isolated from infiltrated leaves as the established protocol (Yoo et al. 2007). Briefly, *N. benthamiana* leaves were cut into 0.5- to 1-mm leaf strips and transferred to prepared enzyme solution (20 mm MES [pH 5.7] containing 1.5% cellulase R10, 0.4% macerozyme R10, 0.4 m mannitol, 20 mm KCl, and 10 mm CaCl_2_). After 30 min of vacuum using a desiccator in the dark, the leaf strips were continued to be digested overnight. W5 solution (2 mm MES [pH 5.7] containing 154 mm NaCl, 125 mm CaCl_2_, and 5 mm KCl) was added to the mixture before being filtered with the 75-mm nylon mesh. Protoplasts were collected by centrifuge and resuspended with W5 solution. The fluorescence of the fusion proteins was examined using a Leica TCS SP5 laser scanning confocal microscope equipped with an excitation wavelength of 488 and 500 to 550-nm band pass filters for GFP and 650- to 710-nm band pass filters for chlorophyll.

### Generation of Arabidopsis transgenic plants

The open reading frame regions (with the stop codon) of *AtPPA-AT* and *AtPPA-AT-C* were amplified using gene-specific primers (Supplementary Table S1) and then ligated into gateway entry vector pENTR/TOPO (Thermo Fisher Scientific). A synonymous mutation in the second 3′ splice site was introduced to *AtPPA-AT* to avoid the alternative splicing for producing solely plastidial proteins (*AtPPA-AT_nas_*) using Q5 site-directed mutagenesis kit (New England Biolabs). To generate the binary vector under the control of *AtPPA-AT* native promoter, the 1,825-bp *AtPPA-AT* native promoter was PCR-amplified with primers. The resulting PCR product and gateway destination vector pK7FWG2 were digested with SacI and SpeI. Two resulting fragments were ligated with T4 ligase. The generation of native promoter by replacing the 35S was confirmed by sequencing. The coding regions of *BigE6*, *B*i*gE6Like*, *AtPPA-AT*, *AtPPA-AT-C*, and *AtPPA-AT_nas_* subcloned into the gateway entry vector pENTR/TOPO were recombined with modified pK7FWG2 vector (*AtPPA-AT* promoter) with the kanamycin selection marker. The recombinant constructs were introduced into *A. tumefaciens* GV3101 and then transformed into *atppa-at-2/+* mutant lines using the floral dip method as described (Clough and Bent 1998). Transgenic plants were screened with kanamycin resistance on MS medium.

### Statistical analysis

The statistical analysis used in this study is summarized in Supplementary Data Set 4.

### Accession numbers

Protein sequences used in this research can be retrieved from Phytozome database with the following accession numbers: *Populus trichocarpa* 1 (Potri.005G079200), *Populus trichocarpa* 2 (Potri.T079800), *Populus trichocarpa* 3 (Potri.007G088400), *A. thaliana* (AT2G22250), *S. lycopersicum* (Solyc04g054710), *Cucumis sativus* (Cucsa.097510), *S. bicolor* 1 (Sobic.009G149400), *S. bicolor* 2 (Sobic.003G370800), *Brachypodium distachyon* 1 (Bradi2g24300), *B. distachyon* 2 (Bradi2g56330), *O. sativa* (LOC_Os01g65090), *Physcomitrium patens* (Pp3c1_16910), *Marchantia polymorpha* (Mapoly0058s0104), *G. max* 1 (Glyma.05G181000), *G. max* 2 (Glyma.11G238300), *G. max* 3 (Glyma.08G138800), and *G. max* 4 (Glyma.11G238200). *Petunia hybrida* (ADM67557.1) and *Selaginella moellendorffii* (PTQ37338.1) were obtained from NCBI.

## Supplementary Material

koaf067_Supplementary_Data

## Data Availability

RNAseq data for transcriptome analyses are deposited in the National Center for Biotechnology Information (NCBI) SRA database under project PRJNA368967.

## Supplementary data

The following materials are available in the online version of this article.

**Supplementary Figure S1.** Three independent *bige6* mutant alleles have indistinguishable phenotypes

**Supplementary Figure S2.** Independent *bige6* mutant alleles alter expression, transcript structure, and/or protein stability.

**Supplementary Figure S3.** Sequence alignments of BIGE6 and BIGE6LIKE with other representative Class Ib aspartate aminotransferases.

**Supplementary Figure S4.** The expression data associated with *BigE6*, *BigE6Like*, and *CM2*.

**Supplementary Figure S5.** The AtPPA-AT was localized in the cytosol and plastids.

**Supplementary Table S1.** Primer list.

**Supplementary Data Set 1.** DEGs in bige6 mutant embryo and endosperm sampled at 20 DAP.

**Supplementary Data Set 2.**  *PPA-AT* gene model analysis.

**Supplementary Data Set 3.** Amino acid sequences used for the phylogenetic tree.

**Supplementary Data Set 4.** Statistical analysis.

## Dive Curated Terms

The following phenotypic, genotypic, and functional terms are of significance to the work described in this paper:

ADT6 Gramene: AT1G08250
ADT6 Araport: AT1G08250

ADT2 Gramene: AT3G07630
ADT2 Araport: AT3G07630

TIR Gramene: AT1G72930
TIR Araport: AT1G72930

ADT1 Gramene: AT1G11790
ADT1 Araport: AT1G11790

ADT4 Gramene: AT3G44720
ADT4 Araport: AT3G44720

ADT5 Gramene: AT5G22630
ADT5 Araport: AT5G22630

IVD Gramene: AT3G45300
IVD Araport: AT3G45300

ADH1 Gramene: AT1G77120
ADH1 Araport: AT1G77120

At2G22250 Gramene: AT2G22250
At2G22250 Araport: AT2G22250
