# Supplementary material for: Maize big embryo 6 reveals roles of plastidial and cytosolic prephenate aminotransferases in seed and plant development
Source: Plant Cell. 2025 Jun 6;37(6):koaf067. doi: 10.1093/plcell/koaf067 (PMC12142466; doi:10.1093/plcell/koaf067)
Supplement: koaf067_Supplementary_Data [file koaf067_supplementary_data.zip › bige6 supplementary figures and tables.pdf]

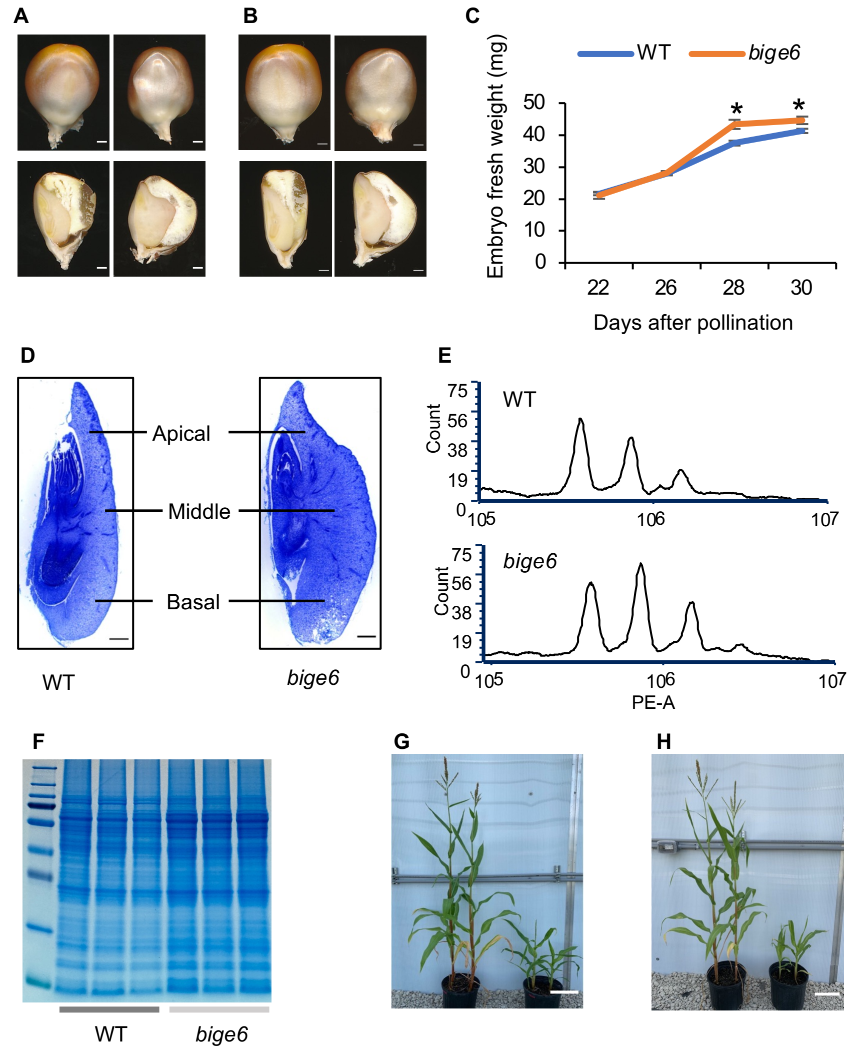

Supplementary Figure S1. Three independent *bige6* mutant alleles have indistinguishable phenotypes. (**A**) and (**G**) Wild type (WT) (left) and *bige6-umu2* (right) kernel and plant phenotypes. (**B**) and (**H**) WT (left) and *bige6-umu3* (right) kernel and plant phenotypes. Scale bar indicates 1 mm in (A) and (B). Scale bar indicates 20 cm in (G) and (H). (**C**) Fresh weights of excised embryos of WT (blue) and *bige6-umu1* mutant (orange). Embryo genotypes were confirmed by PCR with genomic DNA prepared from corresponding endosperms at 22 and 26 days after pollination (DAP). Values are means ± SE. *Differences with t-test $P<0.05$. $n = 10$. (**D**) Representative toluidine blue stained WT and *bige6-umu1* embryos. Lines shown indicate the apical (upper), middle (middle) and basal (lower) region of scutellum. Scale bar = 500 µm. (**E**) The cell ploidy levels of WT (left) and *bige6-umu1* mutant (right) embryos. Three independent embryos for each genotype were mixed and used for extraction of nuclei. Scale bars indicate 1 mm. (**F**) SDS-PAGE analysis of non-zein proteins extracted from mature WT and *bige6* kernels.

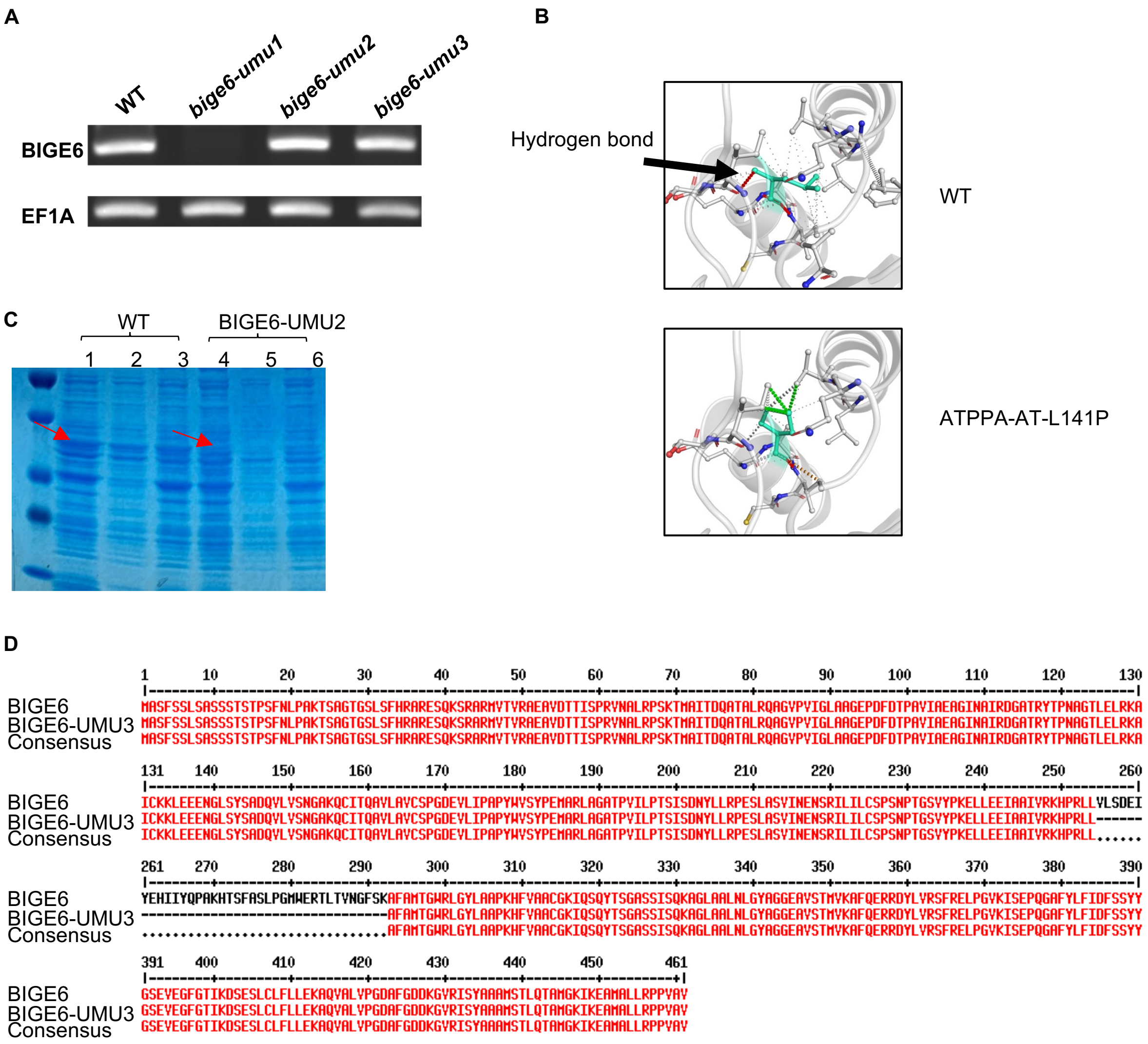

Supplementary Figure S2. Independent *bige6* mutant alleles alter expression, transcript structure and/or protein stability. (**A**) RT-PCR showing the transcription of three *bige6* alleles. EF1A, elongation factor-1 alpha. (**B**) Prediction of Interatomic Interactions of the *bige6-umu2* using DynaMut web server. Dotted red line indicated the hydrogen bond interaction. The light green showed the amino acid substitution, in the upper panel indicating amnio acid Leucine and in the lower panel indicating proline. (**C**) SDS-PAGE analysis of BIGE6 and BIGE6-UMU2 protein extracted from *E. coli*. Line 1 and 4 represented the protein crude extract. Line 2 and 5 indicated supernatant proteins. Line 3 and 6 indicated the inclusion body. (**D**) Sequence alignment between BIGE6 and BIGE6-UMU3 showing the truncated region of BIGE6-UMU3.

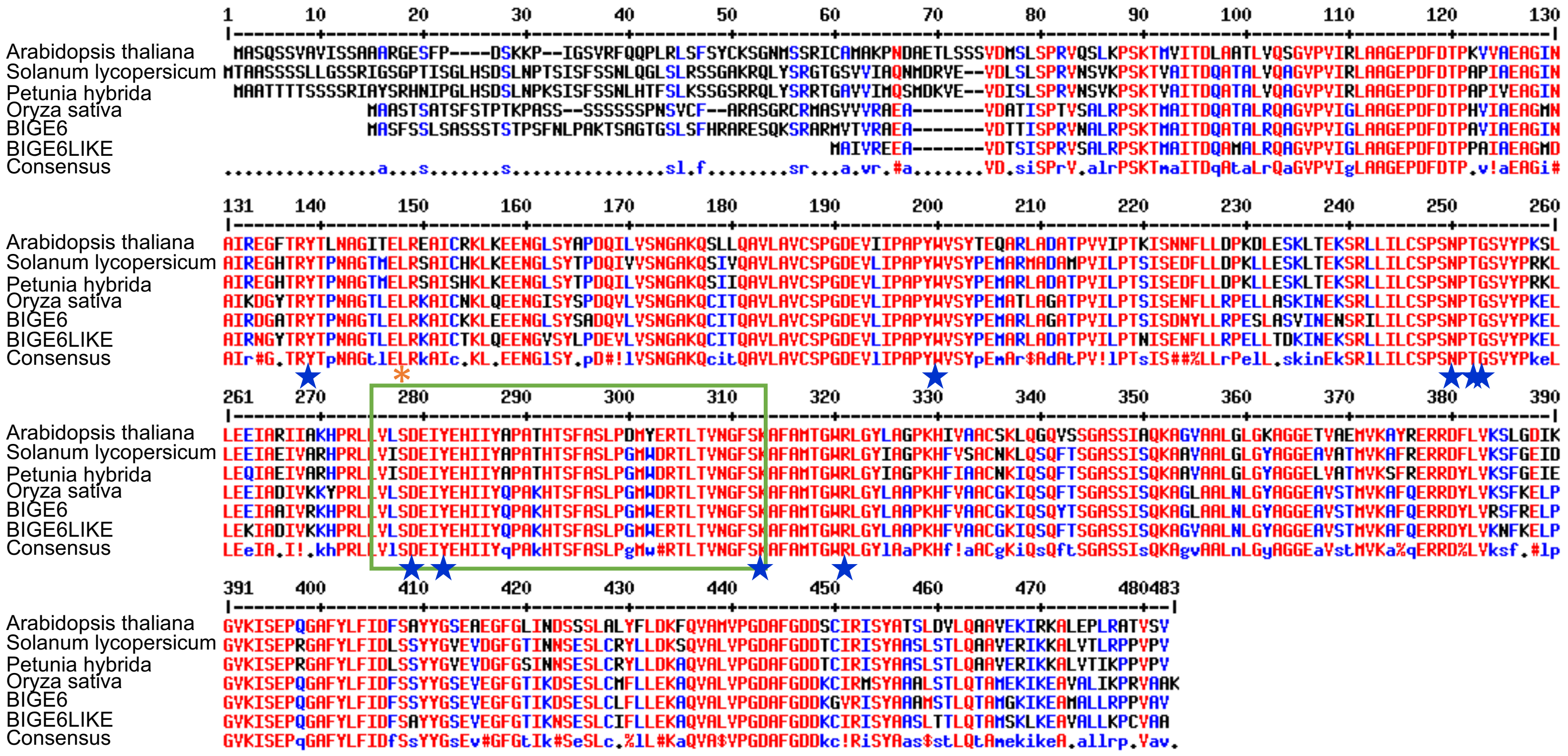

Supplementary Figure S3. Sequence alignments of BIGE6 and BIGE6LIKE with other representative class Ib aspartate aminotransferases. The alignment was performed with the online tool MultAlin (http://multalin.toulouse.inra.fr/multalin/). An orange asterisk indicates the single amino acid substitution at the conserved leucine (L121P) in *bige6-umu2*. A green box denotes the deleted sequences in *bige6-umu3*. Blue stars indicate conserved amino acid residues involved in aspartate aminotransferase activity (Maeda et al., 2011).

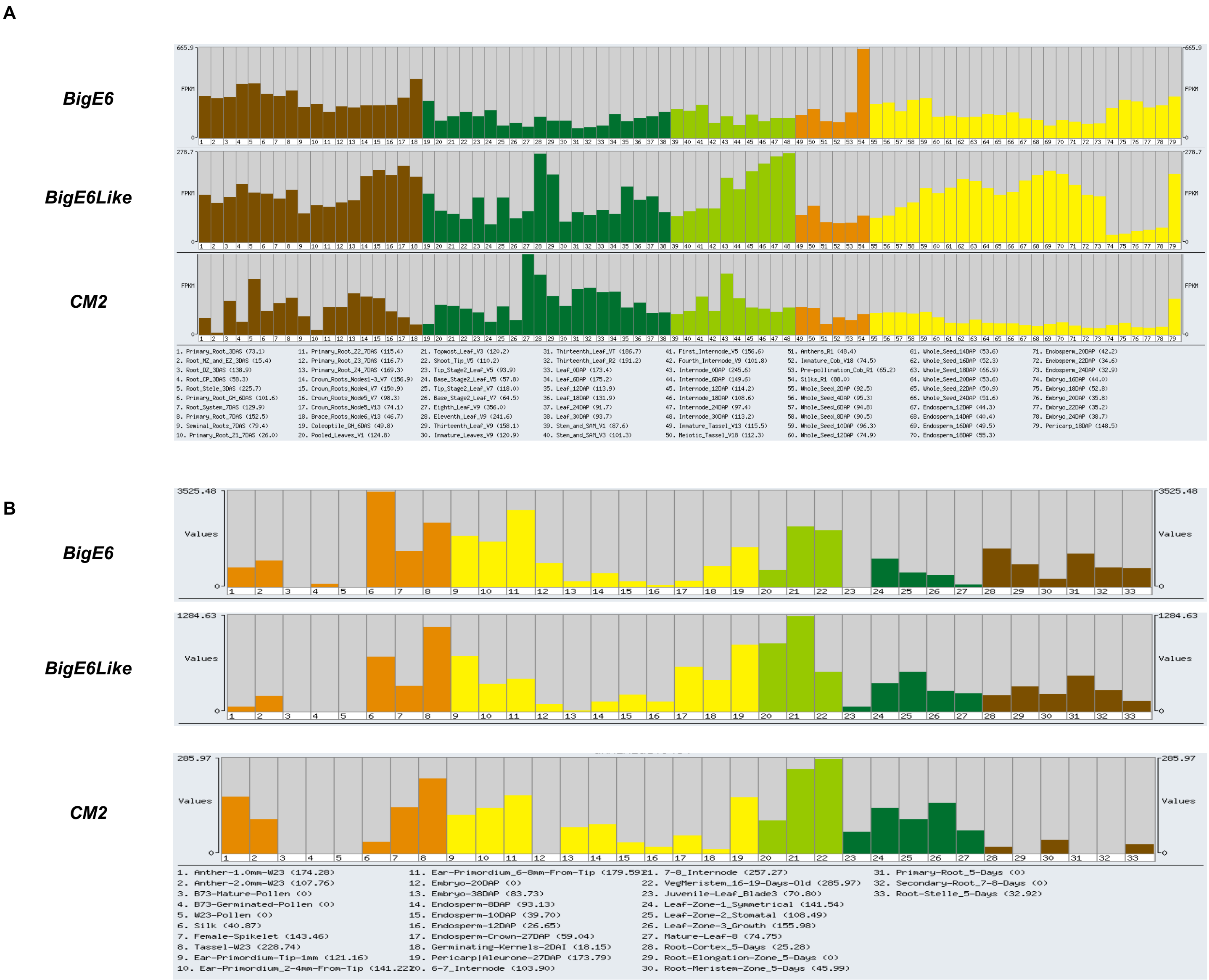

Supplementary Figure S4. The expression data associated with *BigE6*, *BigE6Like* and *CM2*. Transcription (A) and Proteome (B) profiles of *BigE6*, *BigE6Like* and *CM2* genes in maize tissues. Images were from MaizeGDB (B73 RefGen_v3) (https://www.maizegdb.org/).

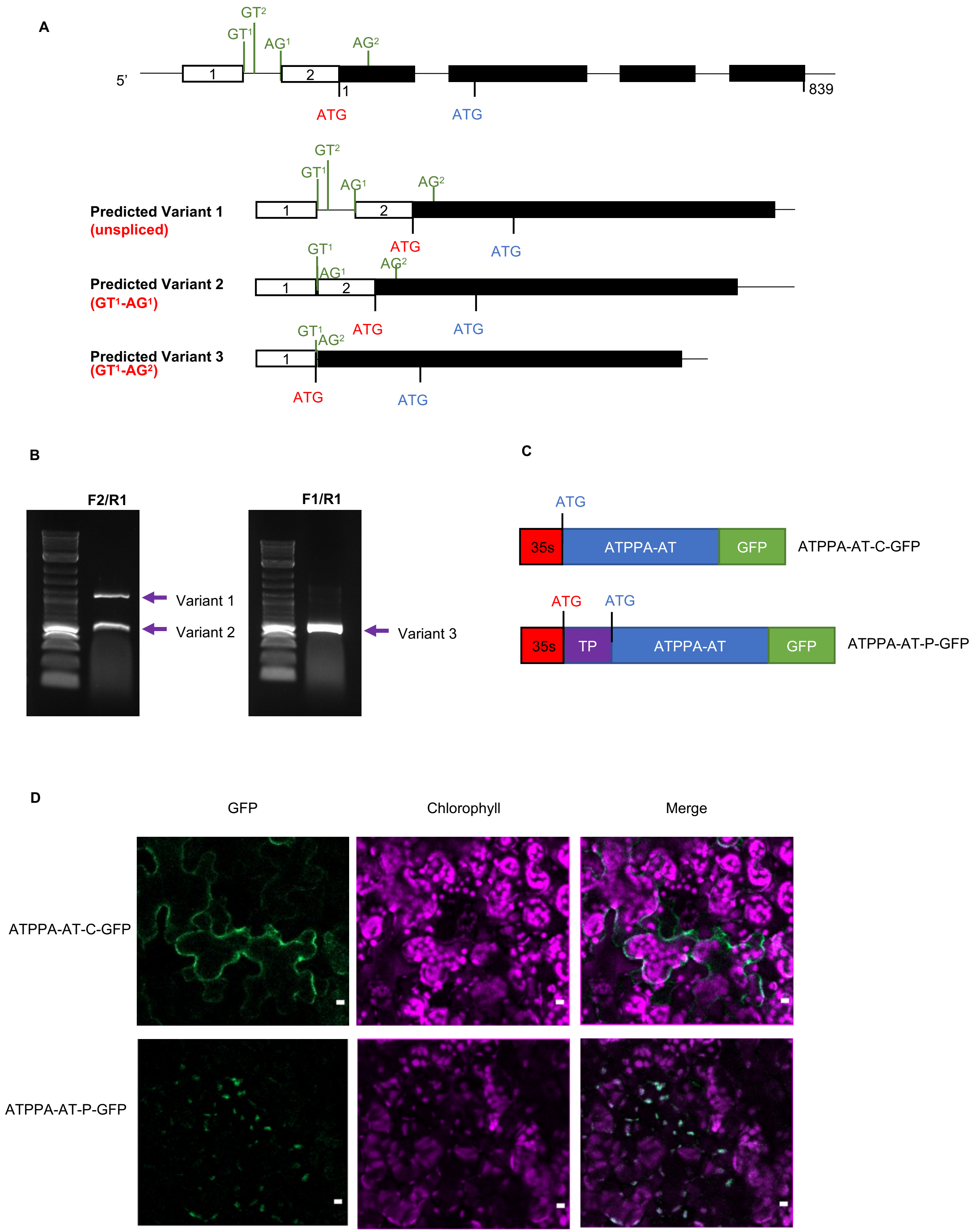

Supplementary Figure S5. **The AtPPA-AT was localized in the cytosol and plastids.** (**A**) Schematic structure of *AtPPA-AT* pre-mRNA and predicted *AtPPA-AT* spliced variants. GT^1, GT^2, AG^1, and AG^2 indicate potential alternative splice sites. The translation start codons are shown in red for plastidial PPA-AT and blue for cytosolic isoforms, respectively. Black boxes and white boxes represent coding exons and untranslated regions (UTRs), respectively. The thin gray lines between boxes represent introns. (**B**) Alternative *AtPPA-AT* variants detected with Arabidopsis root RT-PCR. (**C**) schematic diagram depicting the *AtPPA-AT* constructs. TP, transit peptide. (**D**) Subcellular localizations of AtPPA-AT-GFP fusion proteins. Various *AtPPA-AT* constructs were transient expressed in *N. benthamiana* leaves and analyzed by confocal laser microscope. Chlorophyll (Magenta) is the plastid marker. Scale bars = 10 μm. TP, transit peptide. GFP, green fluorescent protein.

Supplementary Table S1. Primer list

Primers	Sequence (5'→3')	Purpose
**BigE6-GT-F**	GGTCTGAGGTGGAAGGTTTTGGTAC	Mutant genotyping
**BigE6-GT-R**	ACCAAAGCCAAATGACAGCAGCAAC	
**Tir6**	AGAGAAGCCAACGCCAWCGCCTCYATTTCGTC	
**BigE6Like-F3**	AGCAGCTAGCAGCCGCCTCCTCGTCAG	Gene cloning
**BigE6Like-R2**	TGCAATTTAGTGATCTTGCTACGCAGC	
**BigE6-F2**	CAACCCAGCAGCAGCAGGCGCAGCCAG	
**BigE6-R6**	TTTGGTCCACAACTGATTAGCGATACG	
**BigE6-BamHI**	CGGGATCCCATGGCCAGGATGGTGACGGTGCG	
**BigE6-XhoI**	CCCTCGAGCTTAAACGGCAACAGGGGGCC	
**BigE6Like-BamHI**	CGGGATCCCATGGCGATTGTGCGGGAGGA	
**BigE6Like-XhoI**	CCCTCGAGCCTACGCAGCAACACAGGGCT	
**BigE6-GFP-F**	CACCATGGCCTCCTTCTCCTCCCT	Transgenes
**BigE6-GFP-R**	AACGGCAACAGGGGGCCTGA	
**BigE6-GFP-T2-F**	CACCATGGTGACGGTGCGGGCGGA	
**BigE6Like-GFP-F**	CACCATGGCGATTGTGCGGGAGGA	
**BigE6-ike-GFP-R**	CGCAGCAACACAGGGCTTGA	
**BigE6Like-GFP-TP-F1**	CCAGGATGGCGATTGTGCGGGAGGA	
**BigE6Like-GFP-TP-R1**	CCGCACAATCGCCATCCTGGCCCTG	
**AtPPA-AT-OX-F1**	CACCATGGCTTCTCAGAGTTCAGT	
**AtPPA-AT-OX-F2**	CACCATGGCTTCTCAATCTTCAGTCGCCG	
**AtPPA-AT-OX-F3**	CACCATGTCATCTAGAATCTGCGC	
**AtPPA-AT-OX-R1**	TTAAACGGAGACAGTGGCAC	
**AtPPA-AT-GFP-R1**	AACGGAGACAGTGGCACGGA	
**AtPPA-AT-OX-F2**	CACCATGGCTTCTCAATCTTCAGTCGCCG	AtPPA-AT_{nas} mutation
**AtPAT-AS-F1**	CTTAGTTACTTGCTTTCCTG	RT-PCR
**AtPAT-AS-F2**	ATCCCTGGTTCGTTTCTCTT	
**AtPAT-AS-R1**	ATCCATTCTCCTCTTTTAGC	
**BigE6Like-RT-F1**	TGACGATTGTACACTACTCGTCAGATG	
**BigE6Like-RT-R1**	GATTTCTCATTGATCTTGTCGGTAAGC	
**BigE6-RT-F1**	ATCAAGGACTCTGAGTCCCTCTGTCTG	
**BigE6-RT-R1**	TTTGGTCCACAACTGATTAGCGATACG	
**Efa-RT-F1**	ACCATGTATGCCTTACATTCGATGTTC	
**Efa-RT-R1**	CGTCTTGTTGACATCCTTGCAGTTGTC	
**AtPPA-AT-DT1-F0**	TGCCGCAGCTAGTCTAATAACGTTTTAGAGCTAGAAATAGC	CRISPR/Cas9
**AtPPA-AT-DT1-R0**	AACGTGGTTATTCCTACCAAGATCAATCTCTTAGTCGACTCTAC	
**AtPPA-AT-DT1-BsR**	ATTATTGGTCTCGAAACGTGGTTATTCCTACCAAGATCAA	
**U6-26p-F**	TGTCCCAGGATTAGAATGATTAGGC	
**U6-29p-R**	AGCCCTCTTCTTTCGATCCATCAAC	
**U6-29p-F**	TTAATCCAAACTACTGCAGCCTGAC	
**PAtPPA-AT-F3**	CGAGCTCACGAATAGTAGGCCTGGTCC	AtPPA-AT promoter cloning
**PAtPPA-AT-R3**	GGACTAGTTGGAGAAGTTGGGTTGGTGA	
**AtPPA-AT-C9-seq-F**	GGCCAAACCAAATGATGCTGAG	Mutant genotyping
**AtPPA-AT-C9-seq-R**	GACCAAGGAGGCGTTTTATGCT	
**AtPPA-AT-C9-F1**	GCAGGTATTACAGAACTCAG	
**AtPPA-AT-C9-R1**	GGAGAACAAACTGCAAGCAC	
**AtPPA-AT-C9-R2**	AGAGACAGAACATGGCAAAACC	
